# Frequency of extractable nuclear antigen seropositivity among individuals seronegative for antinuclear antibodies on indirect immunofluorescence: a systematic review and meta-analysis

**DOI:** 10.3389/fimmu.2026.1745828

**Published:** 2026-04-28

**Authors:** Tarak Dhaouadi, Imen Zamali, Ahlem Ben Hmid, Mariem Guizani, Awatef Riahi, Taieb Ben Abdallah, Samar Samoud, Yousr Gorgi, Yousr Galai, Imen Sfar, Mélika Ben Ahmed

**Affiliations:** 1 Research Laboratory in Immunology of Renal Transplantation and Immunopathology (LR03SP01), Charles Nicolle Hospital, Tunis El Manar University, Tunis, Tunisia; 2 Faculty of Medicine of Tunis, University of Tunis El Manar, Tunis, Tunisia; 3 Clinical Immunology Department, Pasteur Institute of Tunis, Tunis, Tunisia; 4 Laboratory of Transmission, Control and Immunobiology of Infection, Pasteur Institute of Tunis, Tunis, Tunisia

**Keywords:** antinuclear antibody, connective tissue disease, extractable nuclear antigens, indirect immunofluorescence, meta-analysis, meta-regression

## Abstract

**Background:**

The frequency of extractable nuclear antigen (ENA) seropositivity in patients with negative antinuclear antibodies (ANA) by indirect immunofluorescence (IIF) remains insufficiently characterized. We aimed to estimate the frequency of ENA seropositivity among ANA-IIF–negative individuals (ENA+/IIF−), and to identify associated clinical and methodological determinants.

**Methods:**

A systematic search of PubMed, EMBASE, Web of Science, and Scopus was conducted for studies published up to January 31, 2025. Studies evaluating ENA seropositivity in ANA-IIF–negative patients using HEp-2 or HEp-2000 substrates were included. Meta-analysis was performed using the Freeman–Tukey double arcsine transformation and random-effects models.

**Results:**

Twenty-eight studies, comprising 33 distinct patient groups and 28,552 ANA-IIF–negative samples, were included. The pooled proportion of ENA+/IIF− was 14.1% (95% CI: 10%–18.7%), with substantial heterogeneity (I² = 99%). Subgroup analysis demonstrated significant variation according to clinical indication: 1.4% (95% CI: 1.1%–1.9%) in unspecified indications, 8.1% (95% CI: 6%–10.5%) in suspected connective tissue disease (CTD), and 44.1% (95% CI: 32.3%–54.6%) in confirmed CTD (p < 0.0001). Multivariable meta-regression identified the IIF cut-off dilution, anti-SSA/Ro antibodies, and anti-tRNA synthetase antibodies as significant determinants of ENA+/IIF− frequency.

**Discussion:**

This meta-analysis confirmed that ENA seropositivity is not uncommon in ANA-IIF–negative individuals, and it varies according to clinical context. A negative ANA-IIF result does not reliably exclude CTD, particularly in patients with strong clinical suspicion of CTD. These findings support a targeted, clinically driven ENA testing strategy, especially in conditions such as Sjögren syndrome and inflammatory myopathies.

**Systematic Review Registration:**

https://www.crd.york.ac.uk/PROSPERO/view/, identifier CRD420250654499.

## 1 Introduction

Over the past seven decades, antinuclear antibodies (ANA) have become key biomarkers for diagnosing connective tissue diseases (CTD) (1, 2). However, the term ANA remains imprecise, as it encompasses a broad spectrum of antibodies targeting nuclear, cytoplasmic, and mitotic apparatus antigens (2). Beyond diagnosis, ANA may provide clinically relevant information on disease course, complications, and treatment response (2). Indirect immunofluorescence (IIF), first described by Coons and Kaplan in 1950, remains the reference method for ANA detection (3). It typically relies on HEp-2 cells or HEp-2000 substrates expressing Ro60 and DNA antigens (4). Despite its widespread use, IIF is labor-intensive, requires expert interpretation, and is inherently subjective. Standardization efforts, including the 12th International Workshop on Antibodies and Autoimmunity (Sao Paulo, 2014), have improved harmonization but have not resolved these limitations (5). In addition, IIF lacks specificity, with ANA detected in 5%–25% of healthy individuals depending on technical and demographic factors (6, 7). The increasing demand for ANA testing has driven the development of automated, high-throughput assays, including ELISA, FEIA, CMIA, and MFIA (2, 8). However, their adoption remains debated due to methodological differences from IIF. Two recent meta-analyses reported higher specificity for FEIA [93.6% (95% CI 89.9%–96%) and 94% (95% CI 91%–95%)] and CLIA [86.1% (95% CI 78.3%–91.4%)] compared with IIF [72.4% (95% CI 62.2%–80.7%) and 72% (95% CI 62%–81%)]. In contrast, IIF demonstrated higher sensitivity [86.8% (95% CI 81.8%–90.6%) and 89% (95% CI 84%–93%)] than both FEIA [78.5% (95% CI 71.4%–84.1%) and 73% (95% CI 64%–80%)] and CLIA [85.9% (95% CI 64.7%–95.3%)] (9, 10). Higher specificity reduces false-positive results in healthy individuals, whereas IIF retains superior sensitivity, particularly in systemic lupus erythematosus (SLE) and, to a lesser extent, systemic sclerosis (SSc) (2). Consequently, non-IIF assays are often designed to improve disease-specific detection, such as sclerosis- and myositis-specific immunoassays. In clinical practice, a positive ANA-IIF result is typically followed by antibody subtyping, including anti-dsDNA and extractable nuclear antigen (ENA) testing. Guidelines recommend Farr or CLIFT assays for anti-dsDNA detection and advise confirming ELISA-positive results with these methods (2). ENA antibodies—such as SSA/Ro, SSB/La, RNP, Sm, and Scl-70—were historically detected by double immunodiffusion (DID) but are now assessed using diverse immunoassays with variable performance, such as dot-blot, MFIA, CLIA, and FEIA, among others (2, 11). Because these ENA detection assays exhibit disparate sensitivities and specificities, international guidelines recommend that the method used be indicated on the ANA test results. Therefore, reporting the assay method is essential for accurate result interpretation. Current guidelines recommend ANA subtyping only in patients with positive ANA-IIF and compatible clinical features, an approach that optimizes resource utilization (12, 13). Nevertheless, ENA testing is frequently requested in ANA-negative patients when clinical suspicion remains high, highlighting a gap in diagnostic algorithms. Given the limited data on ENA positivity in ANA-IIF-negative individuals, we conducted a systematic review and meta-analysis to determine the frequency of ENA seropositivity in this population (ENA+/IIF−).

## 2 Materials and methods

### 2.1 Reporting and protocol registration

The current meta-analysis was performed according to the PRISMA guidelines for systematic reviews and meta-analyses (14). This review has been registered on PROSPERO: CRD420250654499, available from: https://www.crd.york.ac.uk/PROSPERO/view/CRD420250654499.

### 2.2 Eligibility criteria

The following selection criteria were adopted:

Inclusion criteria:

- Studies of cross-sectional, case–control, or cohort design.
- Studies assessing ENA subtypes separately in patients with negative ANA by IIF on HEp-2/HEp-2000 substrates.
- Available full texts without language restrictions.

Exclusion criteria:

- ANA detection by methods other than IIF on HEp-2/HEp-2000 substrates.
- The use of an ENA-screen test without determination of ENA subtypes.
- Unavailability of full texts even after contacting the authors.
- Lacking data about total patients with negative ANA that were tested for anti-ENA.
- Case series, narrative or systematic reviews, comments, or meta-analyses.
- If many studies have been carried out using duplicates, only the study with complete data and with the largest sample size was included.

### 2.3 Information sources for study search

An electronic literature search for eligible studies among all papers published prior to 31 January 2025 was conducted through PubMed, Embase, Web of Science, and Scopus databases. The following search strings were used: (((Hep-2 negative) OR (negative ANA) OR (ANA-negative) OR (negative immunofluorescence)) AND ((ENA-positive) OR (positive ENA) OR (positive extractable nuclear antigen))). The literature search was carried out without any language restriction. Manual and/or gray literature searches were not used to supplement the electronic search. A detailed search strategy for each database is available within the **Supplementary Material**.

### 2.4 Study selection

The records were exported from searched databases in order to eliminate duplicates. Studies were screened based on their titles and abstracts. Consequently, studies were assessed and selected based on the data available in full texts that were assessed against eligibility criteria. All studies were independently assessed and evaluated by four reviewers that worked in pairs of two (Tarak Dhaouadi, T.D.; Imen Zamali, I.Z.; Mariem Guizani, M.G.). Two additional investigators (Awatef Riahi, A.R. and Ahlem Ben Hmid, A.B.H.) examined study assessment results.

### 2.5 Data extraction

Data were extracted using a predeveloped form and entered in an Excel datasheet (Appendix). The following variables were recorded independently by two investigators (T.D. and I.Z.): 1) study characteristics: first author, year of publication, study design, department (general lab, immunology lab, clinical medicine department, etc.), time line for data collection, locality setting (community-based or hospital-based), country, ethnicity, and sample size; 2) participant characteristics: mean or median age, gender ratio (M/F), and health condition; and 3) main review variables: context of ANA requests (broad requests, suspected CTD, or diagnosed CTD), ANA detection kit, conjugate (anti-IgG or anti-GAM), IIF cutoff for positive ANA, microscope technology and its light source, ENA testing method, and number of IIF−/ENA+ cases (**Supplementary Table S3, S4**). Differences between the “suspected CTD” and “diagnosed CTD” categories should be interpreted with caution, as a variable proportion of patients initially classified as suspected CTD may ultimately not fulfill CTD diagnostic criteria during follow-up, potentially contributing to interstudy heterogeneity. The frequencies of ANA subtypes, including dsDNA, anti-histone, anti-SSA/Ro (60 or 52), anti-SSB/La, anti-Sm, anti-RNP, anti-CENP-B, anti-Scl-70, anti-Ribosome, and anti-tRNA synthetases Abs, are shown in **Supplementary Material**. Two additional investigators (Imen Sfar, I.S. and Mélika Ben Ahmed, M.B.A.) compared the results of the extracted data for potential discrepancies. In the event of a discrepancy, the two investigators, T.D. and I.Z., reviewed the article concerned by the discrepancy to agree on the final data to be retained.

### 2.6 Quality appraisal

The quality of eligible studies was assessed independently by two reviewers (T.D. and I.Z.) using the JBI critical appraisal tool for systematic reviews of observational epidemiological studies reporting prevalence and cumulative incidence data (15). Studies with a score ≥8 were classified as high-quality reports. Additionally, risk of bias was assessed for each included study through a generic form (Excel spreadsheet) and visualized via the Cochrane ROBVIS online tool (https://mcguinlu.shinyapps.io/robvis/). Four additional independent reviewers (Taieb Ben Abdallah, T.B.A.; Yousr Gorgi, Y.G.; Yousr Galai, Y.Ga.; and Samar Samoud, S.S.) examined the quality assessment results. In the event of a disagreement, the two investigators, T.D. and I.Z., reassessed the article concerned by the inconsistency to reach agreement on the final risk of bias assessment result to be retained.

### 2.7 Statistical analysis

Statistical analysis was carried out using RStudio through the “meta” and “metafor” packages and the OpenMeta-Analyst free software. The pooled proportions with the 95% confidence interval (95% CI) were obtained using the Freeman–Tukey double arcsine transformation as recommended by Munn et al. (15) and Barendregt et al. (16). In fact, this peculiar transformation addresses both the problem of confidence limits falling outside the 0–1 range and the problem of variance instability (15). Random-effects models (DerSimonian–Laird) (17) were applied as recommended by Borenstein et al. (18). Indeed, as long as the eligible studies were carried out in genetically diverse populations, the random-effects model applies (18). Forest plots were generated to display the distribution of effect sizes (pooled frequencies of ENA+/IIF−) across included studies. Sensitivity analyses were carried out to test the stability of the results by omitting sequentially each individual study until no significant change in the overall effect estimate was observed. Between-study heterogeneity was tested by the Q test (significance threshold: 0.1), quantified via *I*^2^ calculation (proportion of true effects variance) and analyzed through the determination of 95% prediction intervals (PI). PI was obtained through the Comprehensive Meta-Analysis (CMA) Prediction Intervals free software. The calculation of the 95% PI was based on the following four items: pooled proportion, upper bound of 95% CI, Tau2 (*τ*²), and the number of included studies. Heterogeneity was classified according to Cochrane cutoffs as follows: 1) *I*^2^ = 0% to 40%: might not be important; 2) *I*^2^ = 30% to 60%: may represent moderate heterogeneity; 3) *I*^2^ = 50% to 90%: may represent substantial heterogeneity; and 4) *I*^2^ = 75% to 100%: considerable heterogeneity. In case of a substantial between-study heterogeneity, outlier (outlying studies) assessment was performed through the generation of the following plots: 1) externally standardized residuals, 2) difference-in-fit standardized statistic (DFFITS), 3) Cook’s distances, 4) covariance ratios, 5) leave-one-out *τ*² estimates, and 6) leave-one-out (residual) heterogeneity test statistic. Subsequently, the heterogeneity was explored for potential sources by subgroup analyses and meta-regressions. Firstly, subgroup analyses were carried out through data stratification by ethnicity [1: Asian, 2: Caucasian, and 3: American (mixed: Caucasians + Afro-Americans + Amerindians)], indication for ENA testing (broad requests, suspected CTD, and diagnosed CTD), ANA detection kit (HEp-2 and HEp-2000), and ENA subtyping method (DID, dot-blot/immunoblot, ELISA, FEIA, and MFIA). Of note, due to limited data on patient race, particularly in studies from the USA, we were unable to categorize the included studies according to race. Secondly, meta-regressions were performed using the following selected variables that could influence the effect size: publication year; patient age; gender ratio (male/female); serum dilution cutoff; and frequencies of dsDNA, anti-histone, anti-SSA/Ro, anti-SSB/La, anti-Sm, anti-RNP, anti-CENP-B, anti-Scl-70, anti-Ribosome, and anti-tRNA synthetase Abs as independent variables. These variables were selected as they might influence both univariable and multivariable models of meta-regression generated to assess the presence of potential confounding factors. Publication bias was assessed by Egger’s test and visualized through the generation of a funnel plot. The *α*-level for statistical significance for all aforementioned analyses was set at 0.05. **Supplementary Material** with additional figures of forest plots generated after the removal of outlying studies is available with the full manuscript. A PRISMA checklist and a PRISMA extension for abstracts checklist are available as **Supplementary Tables S1, S2**.

## 3 Results

### 3.1 Search results and study characteristics

**Figure 1:**
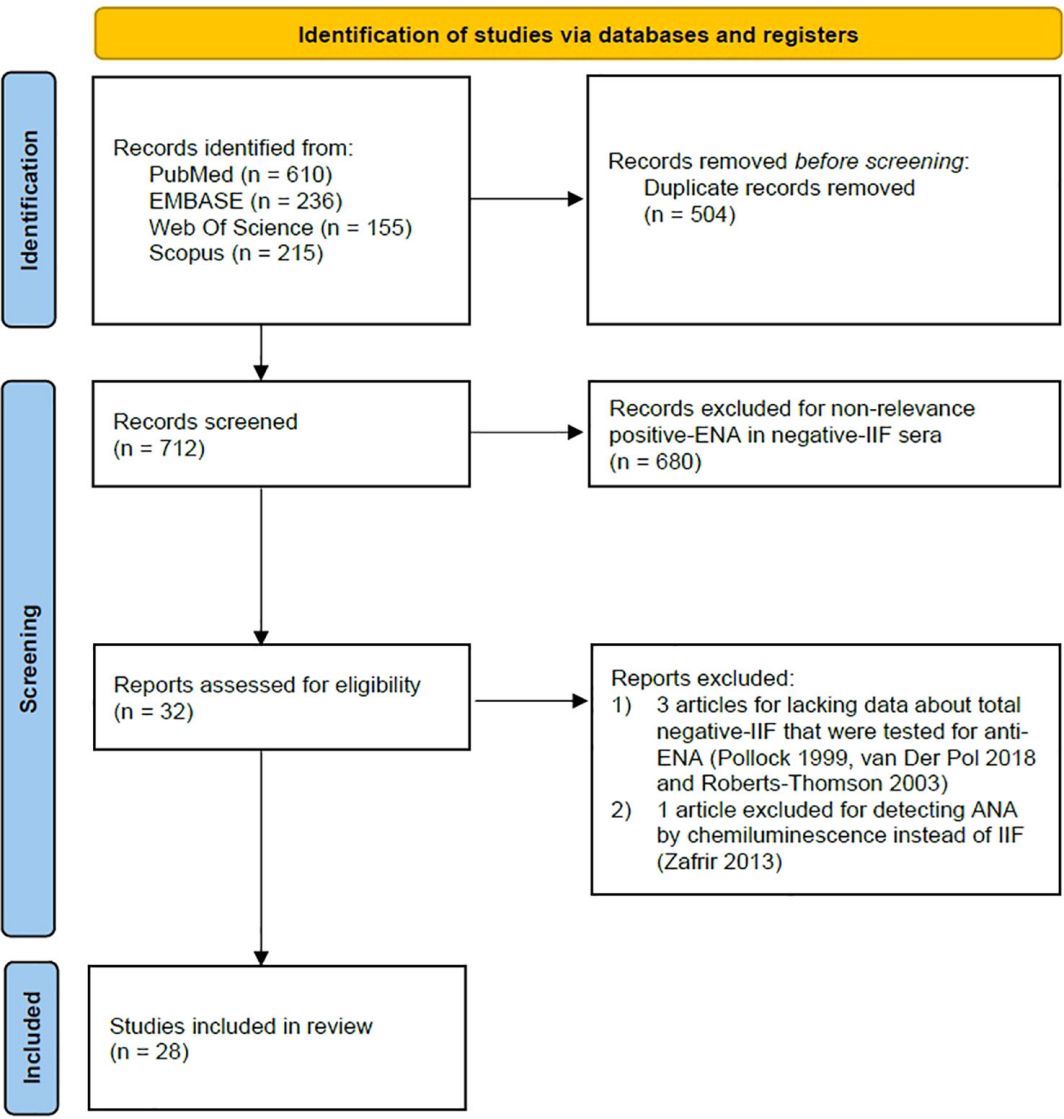
PRISMA flow diagram. Flow diagram of study identification, screening, eligibility, and inclusion according to PRISMA guidelines.

**Figure 2:**
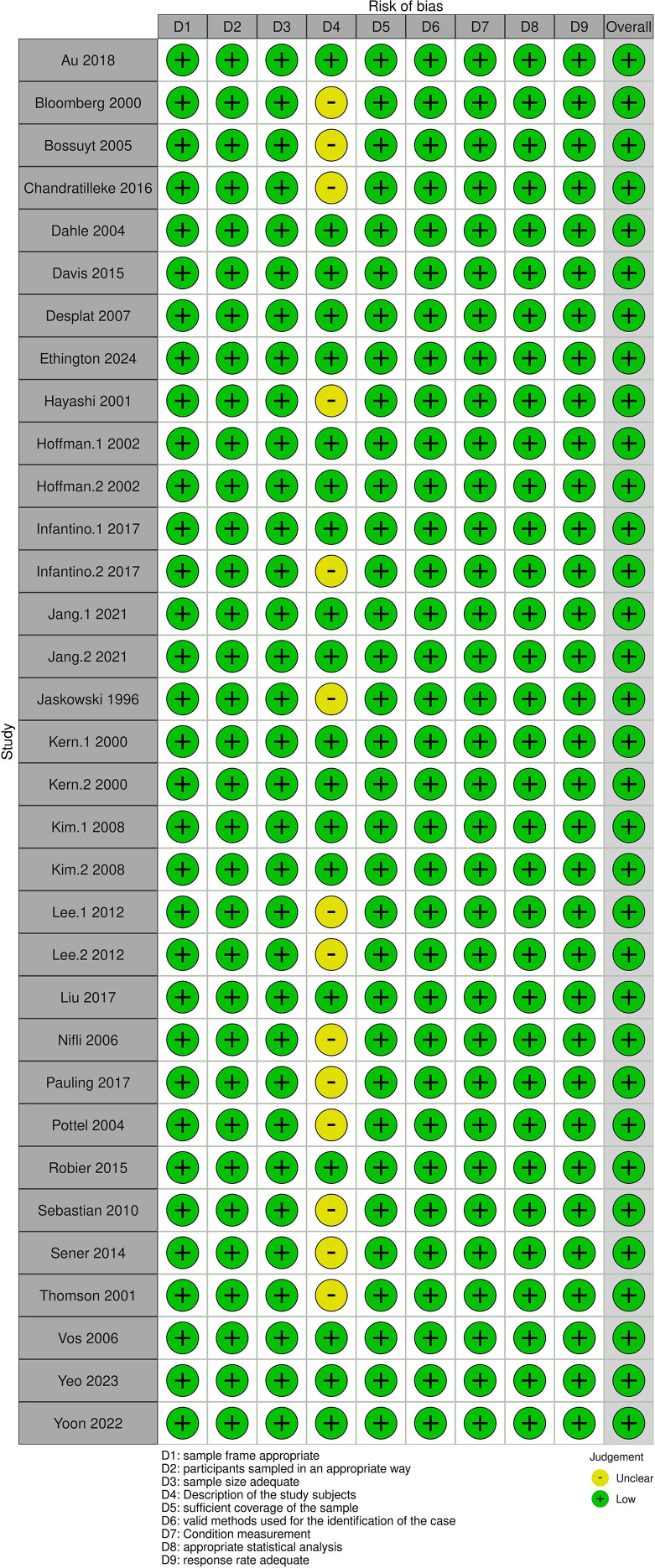
Risk of bias assessment. Summary of risk of bias across included studies using the JBI critical appraisal tool.

A PRISMA flow diagram was generated to depict the study selection process (**Figure 1**). Overall, 28 studies encompassing 33 distinct patient groups [1: Hoffman.1 2002 (HEp-2000) and Hoffman.2 (HEp-2); 2: Jang.1 2021 (ENA test: dot-blot) and Jang.2 (ENA test: FEIA); 3: Kern1. 2000 (site 1: university hospital lab) and Kern.2 2000 (site 2: industrial quality assessment laboratory); 4: Kim.1 2008 (ENA test: dot-blot) and Kim.2 2008 (ENA test: ELISA); and 5: Lee.1 2012 (group 1: diagnosed CTD) and Lee.2 2012 (suspected CTD)] were included with a total of 28,552 IIF-ANA-negative test results, which were represented by one ENA serology test result (19–46). ANA/ENA tests were performed locally in hospital labs (immunology or general) in all included studies. Four studies were excluded due to a lack of data on the total number of participants with negative ANA serology on IIF or because ANA autoantibodies were detected by chemiluminescence instead of IIF (47–50). The characteristics of the included studies and information about ENA laboratory testing are summarized in **Supplementary Tables S3, S4**, respectively. Frequencies of anti-dsDNA, which can be positive in some sera with a negative ANA test, together with ENA subtypes in ANA-negative patients are shown in **Supplementary Table S5**. The JBI critical appraisal quality score results for each included study are depicted in **Supplementary Table S3**. Risks of bias are summarized in **Figure 2**.

### 3.2 Pooled proportion of ENA+/IIF−

**Figure 3:**
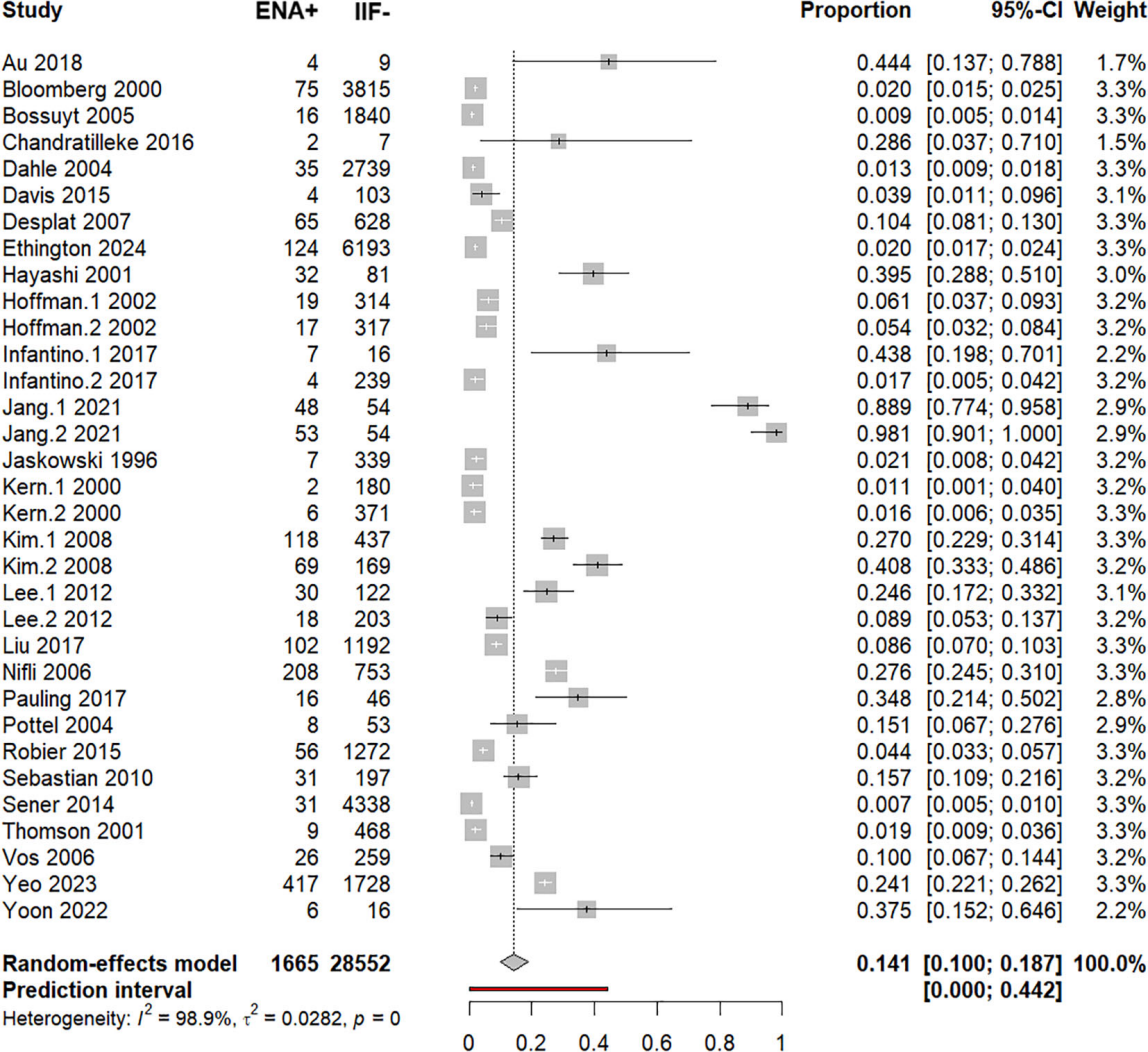
Forest plot of pooled ENA positivity. Pooled proportion of ENA-positive cases among ANA-IIF–negative individuals estimated using a random-effects model.

**Figure 4:**
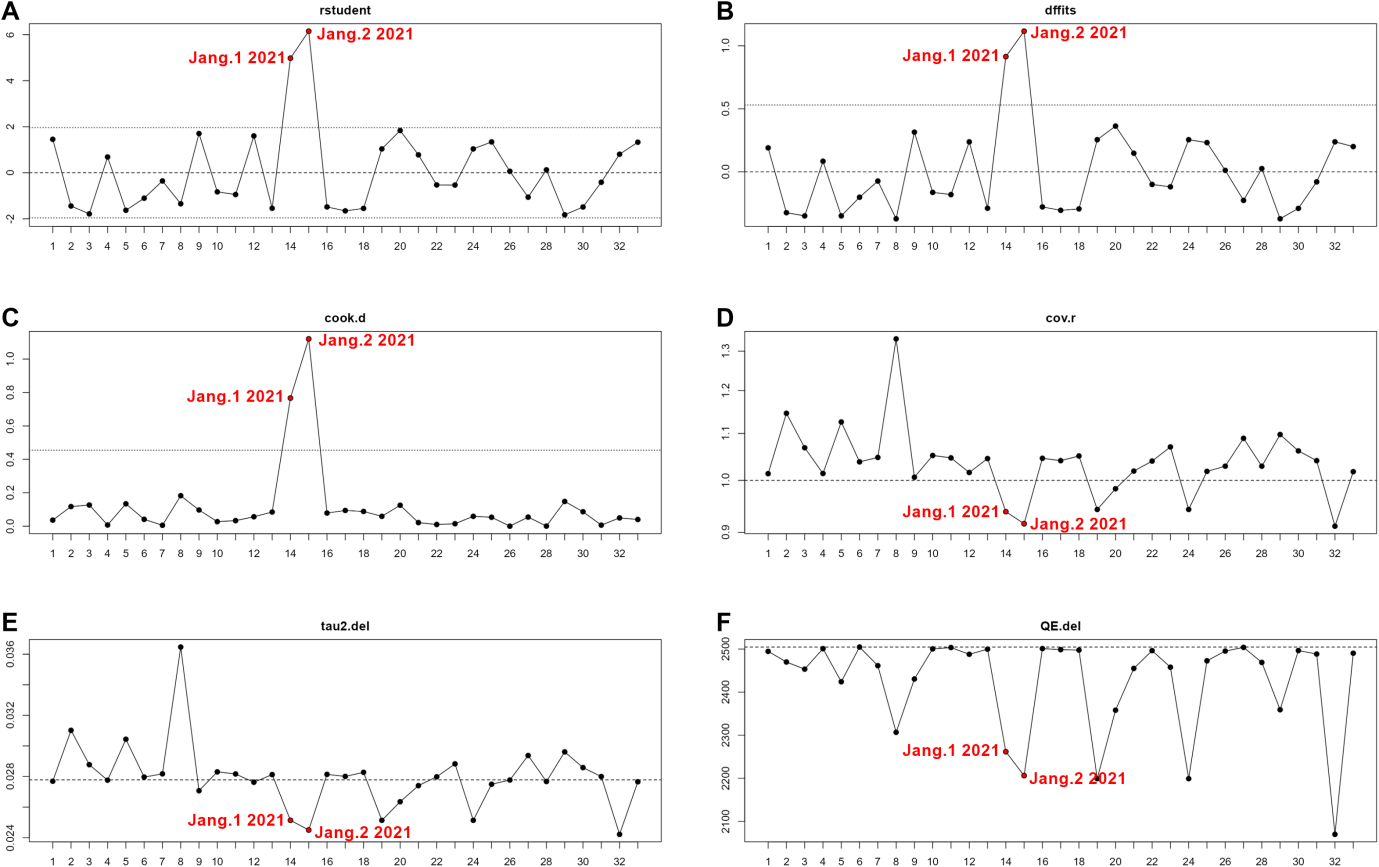
Six-panel line chart summarizing statistical influence diagnostics: **(A–F)** depict rstudent, dffits, cook.d, cov.r, tau2.del, and QE.del, respectively, for thirty-two data points. Red labels highlight “Jang.1 2021” and “Jang.2 2021” as influential outliers across metrics, notably peaking in panels **(A–C)**, with corresponding shifts marked in **(D–F)**.

Combined analysis revealed a pooled proportion of ENA+/IIF− of 14.1% (10%–18.7%) (**Supplementary Table S6**, **Figure 3**). However, there was a substantial amount of between-study heterogeneity, *I*^2^ = 99%, *p* < 0.0001, *τ*² = 0.282, 95% PI = 0%−44.2% (**Supplementary Table S6**, **Figure 3**). Subsequent outlier and influence diagnostics revealed that the two groups (1: ENA test by dot-blot and 2: ENA test by FEIA), extracted from the study of Jang et al. (31), were significantly outlier studies as they significantly increased *τ*² (**Figure 4**). Indeed, all outlier indices showed that both groups, in which ENA+/IIF− frequencies were respectively 88.9% and 98.1%, contributed significantly to the between-study heterogeneity (**Figure 4**). Hence, pooled frequency of ENA+/IIF− after the removal of outlier studies decreased to 10.1% (6.8%–13.9%) (**Supplementary Table S6**, **Supplementary Figure 1**). Nevertheless, the between-study heterogeneity remained significant despite removing the outlier studies (*I*^2^ = 99%, *p* < 0.0001, *τ*² = 0.230, 95% PI = 0%–35.1%).

### 3.3 Subgroup analysis by ethnicity

**Figure 5:**
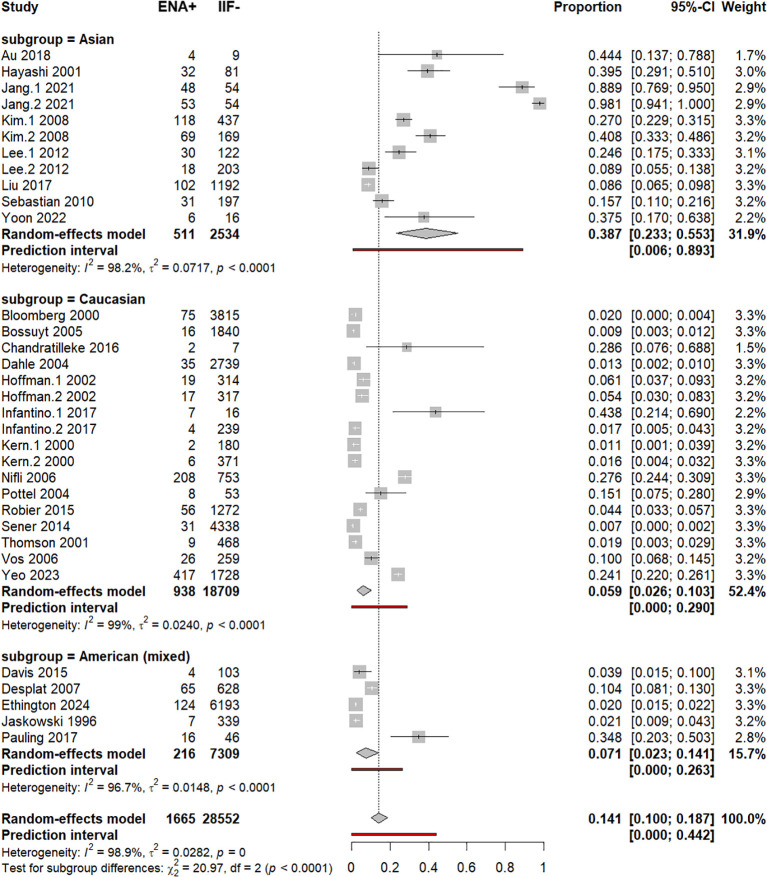
Subgroup analysis by ethnicity. Forest plot of pooled ENA positivity stratified by ethnicity (Asian, Caucasian, American) using random-effects models.

Subgroup analysis by ethnicity and univariable meta-regression showed a significantly higher frequency of ENA+/IIF− cases in Asians [38.7% (23.3%–55.3%)] compared to Americans [7.1% (2.3%–14.1%)] and Caucasians [5.9% (2.6%–10.3%)], *p* < 0.0001 (**Supplementary Table S6, S7**; **Figure 5**). Despite the removal of outlier studies (Jang.1–2021 and Jang.2 2021), ENA+/IIF− remained higher in Asians [24.2% (14.8%–35%)] (**Supplementary Figure 2**). Nevertheless, after adjustment for other covariables, multivariable meta-regression exhibited no interethnic significant difference regarding ENA+/IIF− frequency, *p* = 0.129 (**Supplementary Table S7**).

### 3.4 Subgroup analysis by reason of request of ANA serology test

**Figure 6:**
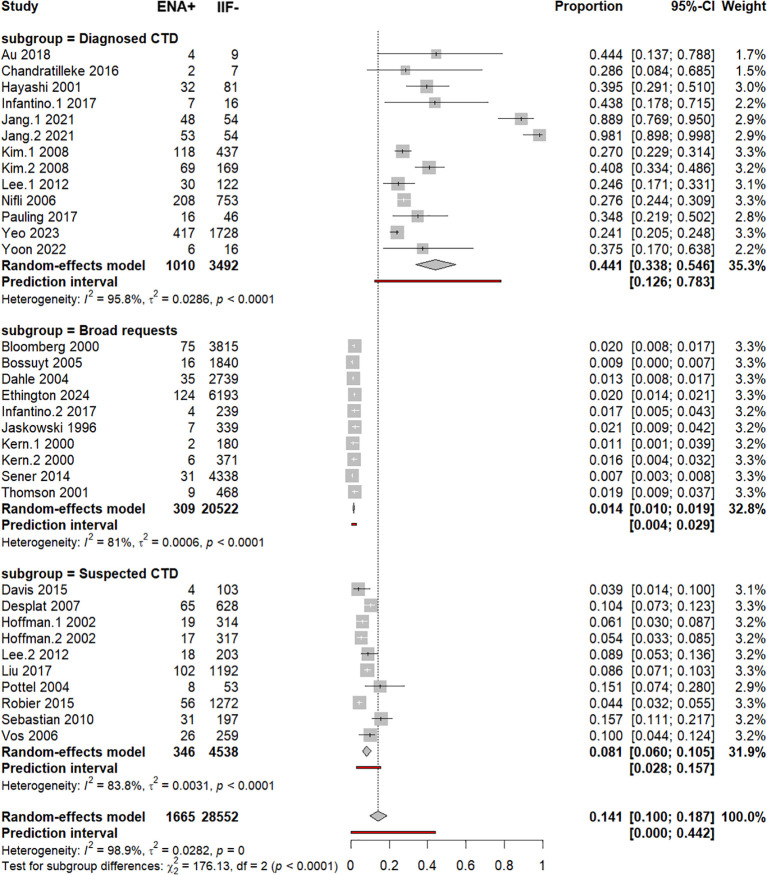
Subgroup analysis by clinical indication. Forest plot of pooled ENA positivity according to clinical indication (broad requests, suspected CTD, confirmed CTD) using random-effects models.

The context of ANA demands was categorized into three main categories as follows: 1) broad requests, 2) suspected CTD, and 3) diagnosed (confirmed) CTD. Subgroup analysis revealed a significantly higher frequency of ENA+/IIF− in case of diagnosed CTD [44.1% (33.2%–54.6%)] than in the suspected CTD subgroup [8.1% (6%–10.5%)] and broad requests subgroup [1.4% (1%–1.9%)], *p* < 0.0001 (**Supplementary Table S6**, **Figure 6**). Even after the removal of outlying studies (Jang.1–2021 and Jang.2 2021), the frequency of the ENA+/IIF− in the sera from confirmed CTD patients [30% (25.8%–34.4%)] remained significantly greater than in the other subgroups (**Supplementary Figure 3**). Univariable and multivariable meta-regressions confirmed that the ENA+/IIF− frequency was significantly higher in confirmed CTD compared to suspected CTD and broad requests settings and in suspected CTD when compared to broad requests (**Supplementary Table S7**).

### 3.5 Subgroup analysis by the ANA detection HEp-2 kit and meta-regression analysis

**Figure 7:**
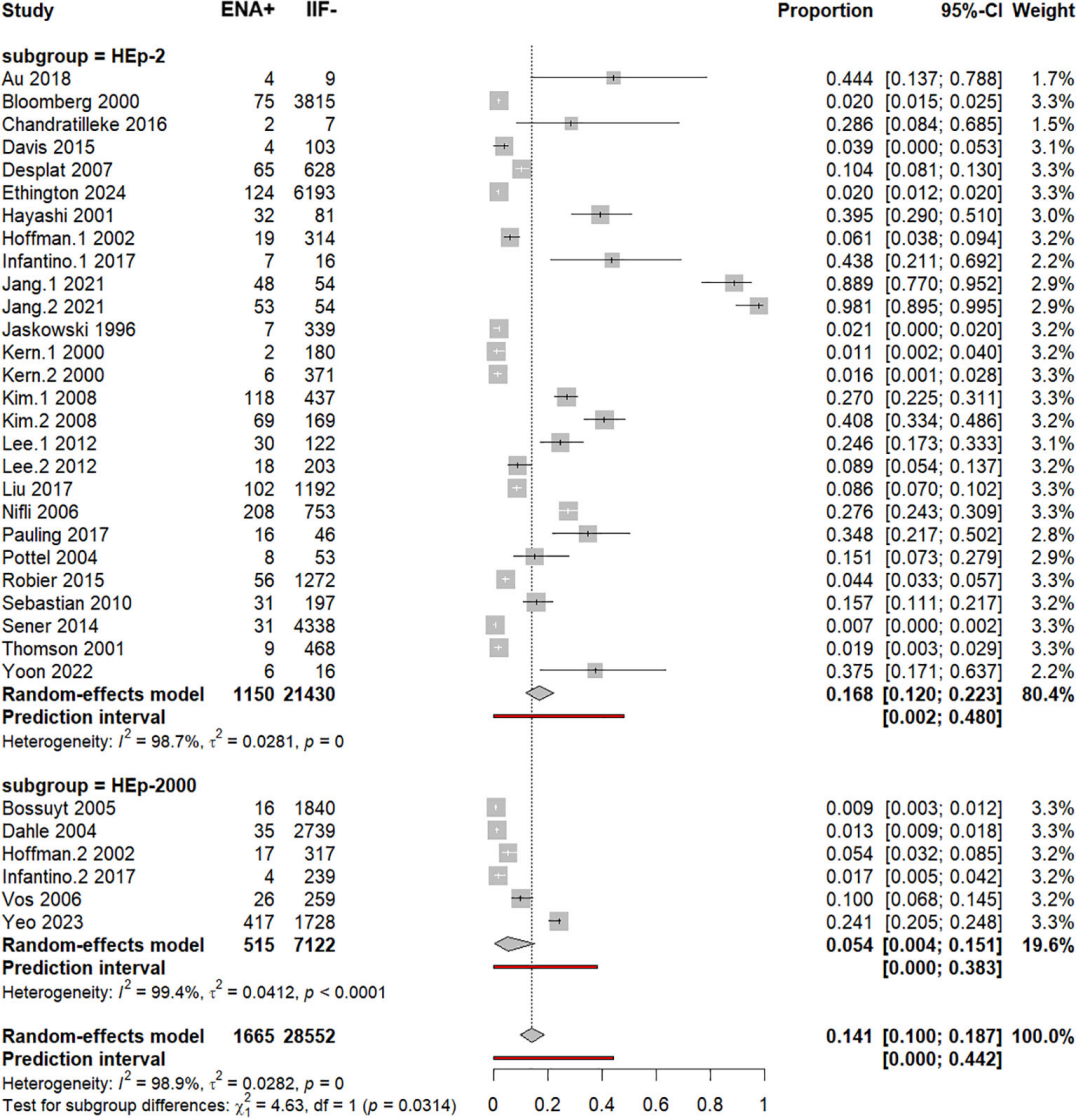
Subgroup analysis by ANA substrate. Forest plot comparing pooled ENA positivity across ANA substrates (HEp-2 vs HEp-2000).

ANA detection kits were categorized as HEp-2 or HEp-2000. ENA seropositivity was significantly higher when ANA were detected by HEp-2 [16.8% (12%–22.3%)] than by HEp-2000 [5.4% (0.4%–15.1%)], *p* = 0.0314 (**Supplementary Table S6**, **Figure 7**). As expected, removal of outlier studies (Jang.1–2021 and Jang.2 2021) decreased the ENA serofrequency in the HEp-2-based negative ANA group to 11.5% (7.8%–15.8%), while there was no outlier study in the HEp-2000 subgroup (**Supplementary Figure 4**). Subsequent univariable and multivariable meta-regression confirmed the significantly higher frequency of ENA+/IIF− cases with the HEp-2 substrate when compared to the HEp-2000 substrate (**Supplementary Table S7**).

### 3.6 Subgroup analysis by the ENA detection method

**Figure 8:**
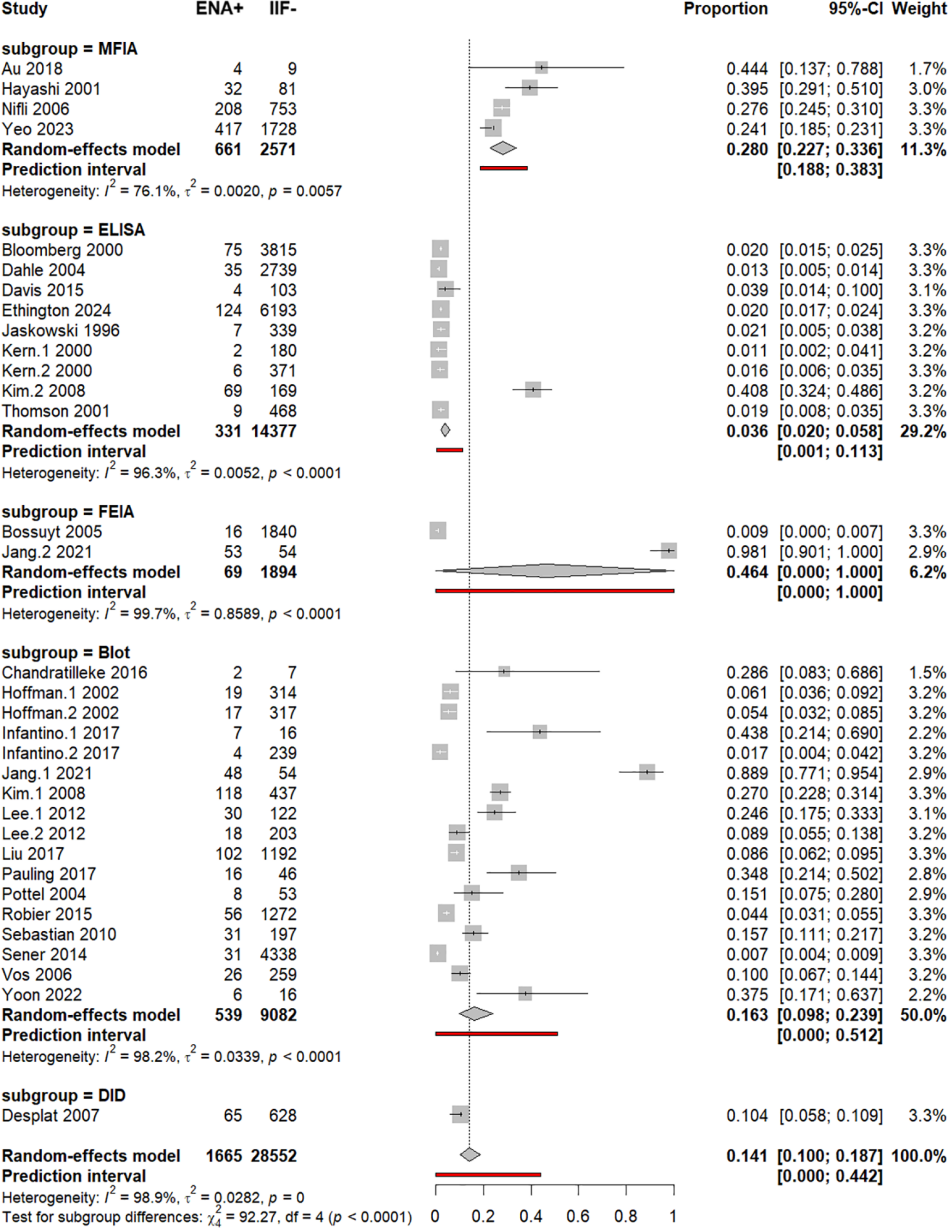
Subgroup analysis by ENA detection method. Forest plot of pooled ENA positivity stratified by assay type (MFIA, ELISA, FEIA, blot, DID) using random-effects models.

Across the included studies, five distinct methods were used for ENA subtyping, i.e., DID (*n* = 1), dot-blot/immunoblot (*n* = 17), ELISA (*n* = 9), FEIA (*n* = 2), and MFIA (*n* = 4). Despite the significant overall *p*-value of the test for subgroup differences, univariable and multivariable meta-regressions did not show any significant correlation between ENA+/IIF− frequency and the ENA detection method (**Supplementary Table S6, S7**; **Figure 8**). Removal of outlier studies (Jang.1–2021 and Jang.2 2021) did not significantly change this finding (**Supplementary Figure 5**).

### 3.7 Meta-regressions for the year of publication and demographic data

**Figure 9:**
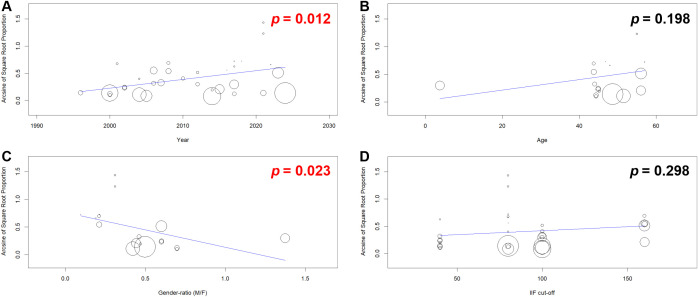
Panel **(A)** is a bubble scatter plot showing a statistically significant (p= 0.012, red font) positive relationship between year and arcsine of square root proportion. Panel **(B)** depicts a non-significant (p = 0.198, black font) association between age and arcsine of square root proportion. Panel **(C)** illustrates a statistically significant (p = 0.023, red font) negative relationship between gender ratio (male to female) and arcsine of square root proportion. Panel **(D)** shows a non-significant (p = 0.298, black font) association between IIF cut-off and arcsine of square root proportion. Each panel features a linear regression line, with data points sized as bubbles.

Univariable and multivariable meta-regressions showed a significant positive correlation of the ENA+/IIF− frequency with the year of publication (**Supplementary Table S7**, **Figure 9A**). Conversely, patients’ age was not correlated with the ENA+/IIF− frequency (**Supplementary Table S7**, **Figure 9B**). Furthermore, while gender ratio (M/F) was significantly and negatively correlated with the ENA+/IIF− frequency in the univariable analysis, multivariable meta-regression did not confirm this peculiar correlation (**Supplementary Table S7**, **Figure 9C**).

### 3.8 Meta-regressions for ANA detection cutoff and ENA subtypes of frequency

**Figure 10:**
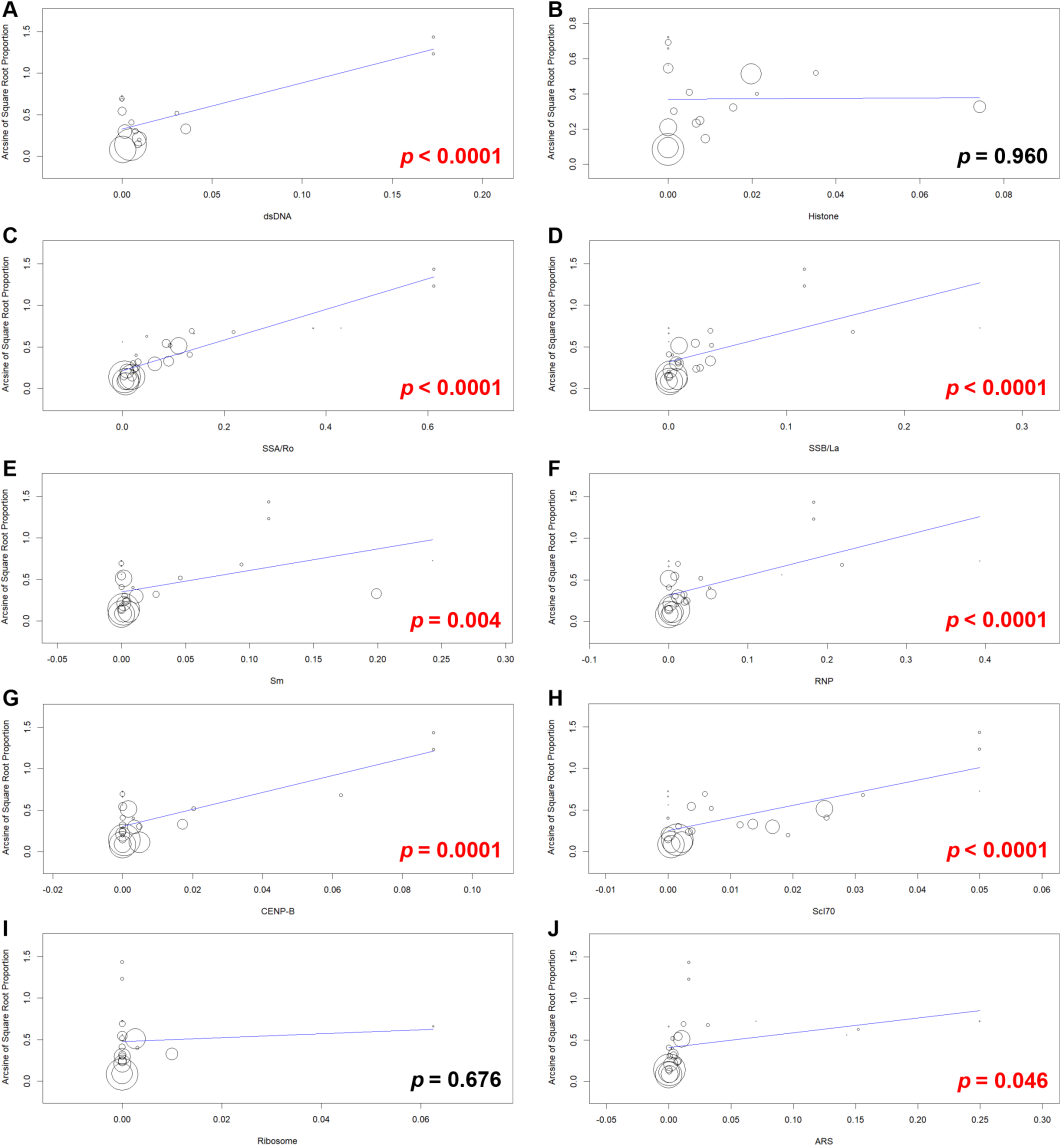
Multipanel figure with ten scatter plots labeled from **(A–J)**, each showing the relationship between antibody measures on the x-axis and the amount of somatic read proportion on the y-axis. Data points are represented by circles of varying sizes, with a fitted trend line on each plot. Statistically significant p-values highlighted in red appear in panels **(A, C–H, J)**, while non-significant p values in black appear in panels B and I. Panels correspond to different autoantibodies: dsDNA, Histone, SSA/Ro, SSB/La, Sm, RNP, Ribosome, CENP-B, Scl70, and ARS.

**Figure 11:**
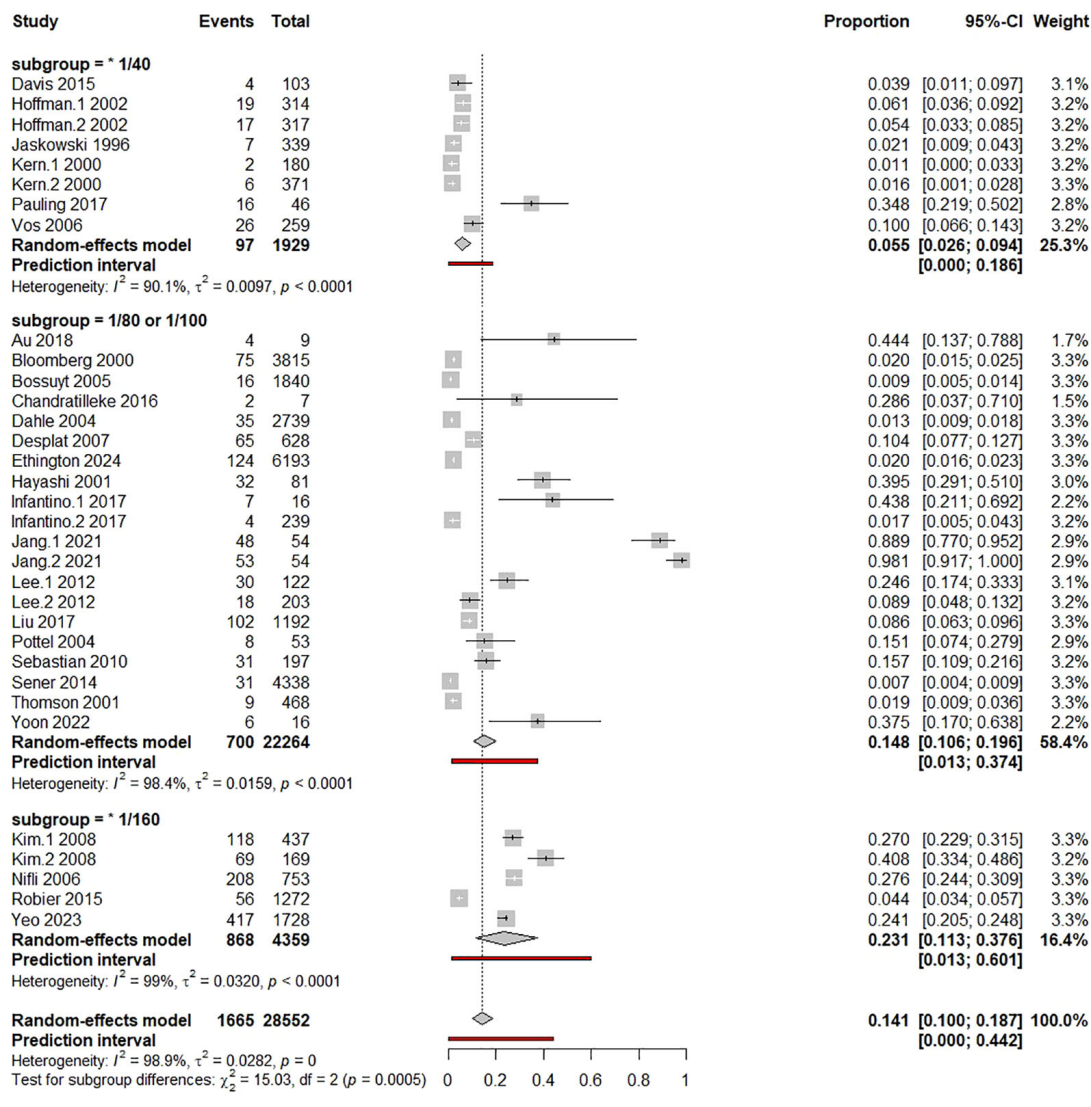
Overall and subgroup pooled estimates. Forest plot summarizing pooled ENA positivity across all studies and predefined subgroups using random-effects models.

Serum dilution cutoff for ANA detection was significantly and positively correlated with ENA+/IIF− frequency in both univariable and multivariable meta-regressions, *p* = 0.014 and *p* = 0.006, respectively (**Supplementary Table S7**, **Figure 9D**). Moreover, univariable meta-regressions revealed significant positive correlations of ENA+/IIF− frequency with dsDNA, anti-SSA/Ro, anti-SSB/La, anti-Sm, anti-RNP, anti-CENP-B, anti-Scl-70, and anti-tRNA synthetase Abs frequencies (**Supplementary Table S7**, **Figure 10**). However, multivariable meta-regression confirmed significant correlations only for anti-SSA/Ro and anti-tRNA synthetase Abs (**Supplementary Table S7**). Subgroup analysis by IIF cutoff and univariable meta-regression showed that higher IIF cutoff (160) was significantly associated with increased ENA+/IIF− cases (**Supplementary Table S6, S7**; **Figure 11**). This significant positive association was confirmed by multivariable meta-regression (**Supplementary Table S7**).

### 3.9 Sensitivity analysis

**Figure 12:**
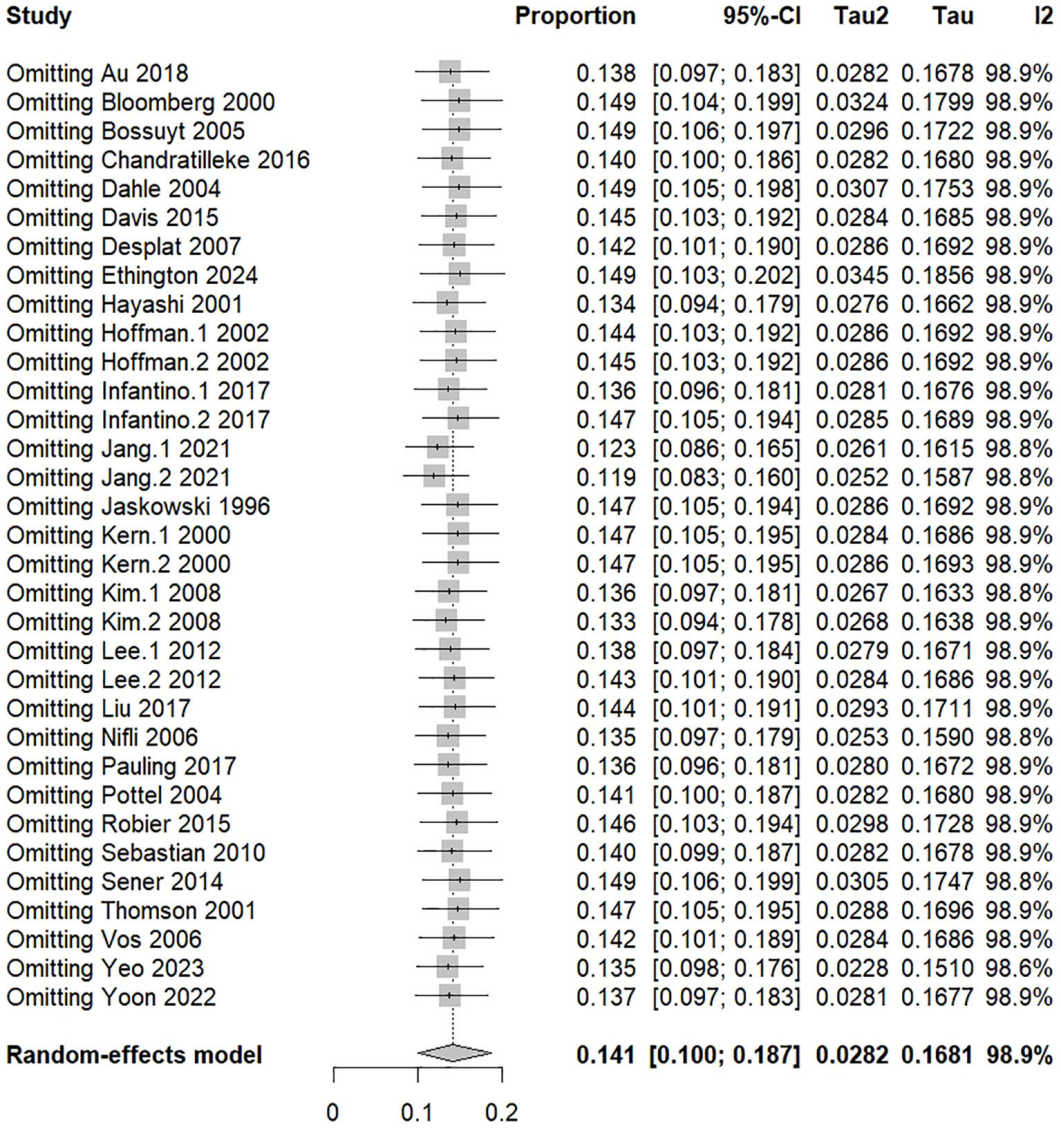
Sensitivity analysis. Leave-one-out analysis illustrating the influence of individual studies on pooled estimates and heterogeneity.

The sensitivity analysis revealed that the overall ENA+/IIF− serofrequency was stable, suggesting a high level of integrity with reliable results (**Figure 12**).

### 3.10 Publication bias

**Figure 13:**
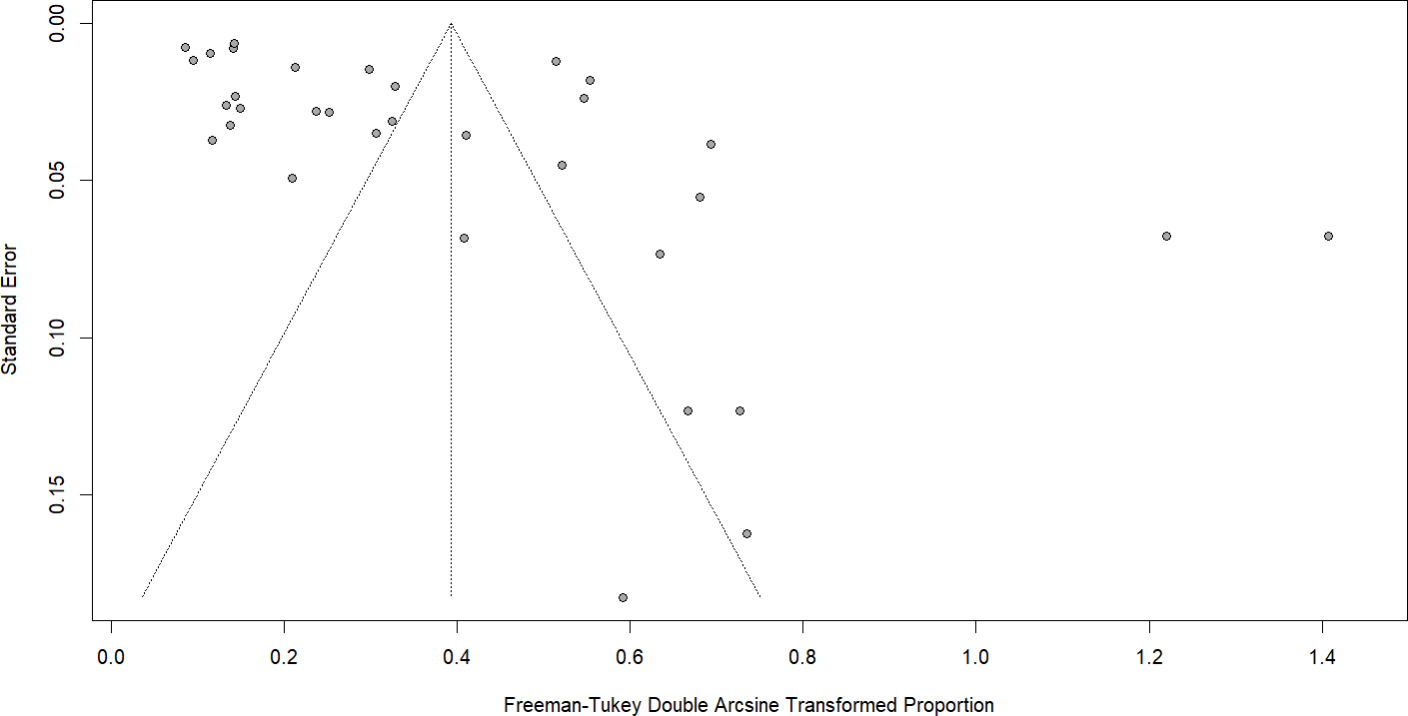
Funnel plot for publication bias. Funnel plot of standard error versus transformed proportions to assess small-study effects and potential publication bias.

The generated funnel plot (**Figure 13**) was found to be overall symmetrical. Despite the high level of between-study heterogeneity, which could cause false-positive result of publication bias assessment, Egger’s regression test yielded a non-significant *p*-value (0.09258), which indicated that the results were not weakened by publication bias.

## 4 Discussion

Previous studies have shown that ENA positivity may occur despite a negative ANA-IIF result, although reported frequencies vary widely (19–46). In all the included studies, testing was performed in hospital laboratories, reflecting real-world clinical practice. In the present systematic review and meta-analysis, ENA seropositivity was observed in 14.1% (10%–18%) of ANA-IIF-negative sera. However, substantial heterogeneity was observed, with frequencies ranging from 0% to 45.5% in 95% of comparable populations. After exclusion of outlier studies (31), the pooled frequency decreased to 10.1% (6.8%–13.9%), but heterogeneity remained very high (*I*² = 99%, 95% PI 0–36%). Importantly, this high heterogeneity indicates that the observed variability reflects true differences in study populations, clinical contexts, and laboratory methods rather than random variation alone. This significantly limits the generalizability of the pooled estimate, which should be interpreted as an average across heterogeneous settings rather than a universal value. Accordingly, the probability of ENA+/IIF− varies substantially depending on patient characteristics, indication for testing, and technical factors. Therefore, subgroup-specific results are more clinically informative than the overall pooled estimate. ENA seropositivity may occur in a clinically relevant proportion of patients with negative ANA-IIF. However, the wide variability across studies is explained by both clinical and methodological factors. Clinical factors include age, sex, genetic background, and, most importantly, the indication for ANA testing. Methodological factors include the type of HEp-2 substrate, IIF cutoff dilution, ENA assay platform, antigen composition, and operator-dependent interpretation of fluorescence patterns, which remain major sources of interlaboratory variability. Among these factors, the clinical context of ANA testing was the main driver of heterogeneity. The highest ENA+/IIF− frequency [44.1% (33.2%–54.6%)] was observed in patients with confirmed CTDs (**Supplementary Table S3**), including SLE (19), idiopathic inflammatory myopathies (IIM) (29), systemic sclerosis (SSc) (38), and mixed CTDs (22, 27, 31, 34, 35, 37, 45, 46). However, the lack of detailed subgroup data prevented further stratification by CTD subtype. In addition, treatment exposure was not reported, although immunosuppressive and biologic therapies may reduce autoantibody detectability. In patients with suspected CTD, the pooled ENA+/IIF− frequency was lower [8.1% (6%–10.5%)] but remained heterogeneous. This indicates that ANA-IIF may yield false-negative results in a non-negligible proportion of clinically suspected cases. This frequency may vary substantially, ranging from approximately 2.2% to 17%, depending on the clinical setting. In studies with broad or non-specific indications, the ENA+/IIF− frequency was low [1.4% (1%–1.9%)], highlighting potential inappropriate test utilization. Although the diagnostic yield is limited, a negative ANA-IIF does not formally exclude ENA positivity or CTD, particularly in early or atypical disease. Methodological variability also contributed substantially to heterogeneity. The ENA+/IIF− frequency was higher with conventional HEp-2 substrates [16.8% (12%–22.3%)] than with HEp-2000 cells [5.4% (0.4%–15.1%)]. The HEp-2000 substrate, transfected with SSA/Ro60 cDNA, may improve sensitivity for anti-SSA/Ro60 detection, although it remains imperfect (51). Indeed, the HEp-2000 substrate was found to detect anti-SSA/Ro60 Abs when missed on conventional immunoassays such as immunoblotting and the standard HEp-2 substrate (51). Nevertheless, this HEp-2000 substrate is not a perfect screen, as it may still miss the detection of anti-SSA/Ro60 in positive sera by other immunoassays (51). Despite improved sensitivity, the International Consensus on ANA Patterns (ICAP) has decided not to include manipulated cellular substrates such as HEp-2000 (52). This decision was based on concerns that the characteristics of HEp-2000 subcultures may have progressively diverged from those of the original HEp-2 cells, with a potential risk of contamination by HeLa cells. In addition, anti-SSA/Ro60 antibodies produce an “atypical speckled” pattern on HEp-2000 cells, which differs significantly from the classical pattern observed on standard HEp-2 cells, namely, the AC-4 (nuclear fine speckled) pattern. Such discrepancies may complicate pattern recognition and interpretation. Despite these limitations, the ICAP consortium acknowledged that HEp-2000 cells may have clinical utility, particularly for the identification of women of childbearing age at risk of pregnancy complications, including congenital heart block and neonatal lupus. Overall, ICAP does not recommend the use of modified substrates such as HEp-2000, primarily due to concerns related to pattern interpretation, substrate variability, and potential contamination (52). The IIF cutoff dilution was another major source of variability. Higher cutoffs improve specificity but reduce sensitivity, whereas lower cutoffs increase sensitivity at the expense of specificity, with ANA positivity observed in up to 25% of healthy individuals (6, 7). Hence, the use of lower dilution cutoff value (i.e., 1/80) might reduce the serodiscordance between ENA and ANA autoantibodies. However, applying lower cutoff values could reduce the specificity of IIF, as the frequency of ANA positivity may increase in healthy subjects. This might be useful to apply lower cutoffs in cases of strong suspicion of CTD. The lack of standardization (cutoffs ranging from 1:40 to 1:200) further contributes to interstudy heterogeneity. No significant effect of the ENA assay platform was observed in the multivariable analysis. However, intermethod variability remains important due to differences in antigen preparation, detection systems (IgG vs. GAM), and positivity thresholds (2). In addition, variability in ENA panels, including non-classical specificities (e.g., Ku, PM-Scl, Mi-2), may increase ENA+/IIF− discordance. A significant positive association between year of publication and ENA+/IIF− frequency was observed, likely reflecting improved assay sensitivity, broader antigen panels, and increased clinical awareness rather than a true epidemiological change. Multivariable meta-regression identified anti-SSA/Ro and anti-tRNA synthetase antibodies as independent contributors to ENA+/IIF− discordance, consistent with the lower sensitivity of HEp-2 IIF for these autoantibodies (2). Particular attention is therefore warranted in patients with suspected Sjögren syndrome and idiopathic inflammatory myopathies and in pregnant women at risk of anti-SSA/Ro-related complications. Several limitations should be considered. Although the search strategy was comprehensive, gray literature was not systematically included, which may have introduced publication bias. The included studies were limited to Caucasian, Asian, and North American populations, restricting generalizability. Incomplete reporting of patient race—particularly in U.S. studies—precluded adjustment, while racial categorization itself may oversimplify heterogeneity. All studies were hospital-based, limiting applicability to screening or low-prevalence settings. Although ENA testing has long been available, relevant data only emerged from 1996 onward. In addition, some subgroup analyses were based on a limited number of studies; for instance, only six studies used the HEp-2000 substrate, and only a few employed methods such as DID, FEIA, or MFIA. This limited the robustness of heterogeneity assessment and the precision of between-study variance *τ*² estimation. The inability to distinguish anti-SSA/Ro60 from anti-SSA/Ro52 antibodies—due to reliance on aggregate data and incomplete reporting—precluded stratified analyses, including evaluation of potential effects related to Ro60 overexpression in HEp-2000 substrates. Additionally, most studies did not report ENA+/IIF− frequencies by CTD subtype, preventing disease-specific comparisons. Key methodological details, including microscope characteristics, were often missing, and several potential confounders (e.g, comorbidities, pre-analytical factors, assay variability, and operator expertise) were not reported. Given the substantial heterogeneity, Egger’s test for publication bias should be interpreted cautiously.

Despite these limitations, this study provides the first comprehensive laboratory-based meta-analysis of ENA+/IIF− frequency. Clinically, a negative ANA by IIF does not exclude the presence of ENA antibodies. However, these findings do not support indiscriminate ENA testing. Instead, testing should be guided by clinical suspicion and interpreted within the appropriate clinical context. A patient-centered, targeted approach remains essential to optimize diagnostic accuracy and avoid unnecessary testing.

## 5 Conclusion

This meta-analysis demonstrates that serodiscrepancies between ANA-IIF and ENA exist in a significant proportion of patients (14.1%), particularly in patients with suspected (8.1%) or confirmed CTDs (44.1%). In patients with strong clinical suspicion of CTD, ENA testing may therefore be justified despite a negative ANA-IIF result. These discrepancies are influenced by both clinical and methodological factors, including HEp-2 substrate, IIF cutoff, and the presence of specific autoantibodies such as anti-SSA/Ro and anti-tRNA synthetases. Overall, a targeted, clinically driven approach remains essential to ensure accurate diagnosis while avoiding unnecessary testing.

## Data Availability

The original contributions presented in the study are included in the article/**Supplementary Material**. Further inquiries can be directed to the corresponding author.

## References

[1] Solomon DH Kavanaugh AJ Schur PH American College of Rheumatology Ad Hoc Committee on Immunologic Testing Guidelines . Evidence-based guidelines for the use of immunologic tests: antinuclear antibody testing. Arthritis Rheum. (2002) 47:434–44. doi: 10.1002/art.10561. PMID: 12209492

[2] Agmon-Levin N Damoiseaux J Kallenberg C Sack U Witte T Herold M . International recommendations for the assessment of autoantibodies to cellular antigens referred to as antinuclear antibodies. Ann Rheum Dis. (2014) 73:17–23. doi: 10.1136/annrheumdis-2013-203863. PMID: 24126457

[3] Coons AH Kaplan MH . Localization of antigen in tissue cells; improvements in a method for the detection of antigen by means of fluorescent antibody. J Exp Med. (1950) 91:1–13. doi: 10.1084/jem.91.1.1. PMID: 15395569 PMC2135948

[4] Meroni PL Schur PH . ANA screening: an old test with new recommendations. Ann Rheum Dis. (2010) 69:1420–2. doi: 10.1136/ard.2009.127100. PMID: 20511607

[5] Chan EK Damoiseaux J Carballo OG Conrad K de Melo Cruvinel W Francescantonio PL . Report of the first international consensus on standardized nomenclature of antinuclear antibody HEp-2 cell patterns 2014–2015. Front Immunol. (2015) 6:412. doi: 10.3389/fimmu.2015.00412. PMID: 26347739 PMC4542633

[6] Li QZ Karp DR Quan J Branch VK Zhou J Lian Y . Risk factors for ANA positivity in healthy persons. Arthritis Res Ther. (2011) 13:R38. doi: 10.1186/ar3271. PMID: 21366908 PMC3132017

[7] Satoh M Chan EK Ho LA Rose KM Parks CG Cohn RD . Prevalence and sociodemographic correlates of antinuclear antibodies in the United States. Arthritis Rheum. (2012) 64:2319–27. doi: 10.1002/art.34380. PMID: 22237992 PMC3330150

[8] Fritzler MJ . The antinuclear antibody test: last or lasting gasp? Arthritis Rheum. (2011) 63:19–22. doi: 10.1002/art.30078. PMID: 20954183

[9] Orme ME Andalucia C Sjölander S Bossuyt X . A hierarchical bivariate meta-analysis of diagnostic test accuracy to provide direct comparisons of immunoassays vs. indirect immunofluorescence for initial screening of connective tissue diseases. Clin Chem Lab Med. (2020) 59:547–61. doi: 10.1515/cclm-2020-0094. PMID: 32352399

[10] Orme ME Andalucia C Sjölander S Bossuyt X . A comparison of a fluorescence enzyme immunoassay versus indirect immunofluorescence for initial screening of connective tissue diseases: systematic literature review and meta-analysis of diagnostic test accuracy studies. Best Pract Res Clin Rheumatol. (2018) 32:521–34. doi: 10.1016/j.berh.2019.03.005. PMID: 31174821

[11] Infantino M Carbone T Brusca I Alessio MG Previtali G Platzgummer S . Current technologies for anti-ENA antibody detection: state of the art of diagnostic immunoassays. J Immunol Methods. (2022) 507:113297. doi: 10.1016/j.jim.2022.113297. PMID: 35690095

[12] Bonaguri C Melegari A Ballabio A Parmeggiani M Russo A Battistelli L . Italian multicentre study for application of a diagnostic algorithm in autoantibody testing for autoimmune rheumatic disease: conclusive results. Autoimmun Rev. (2011) 11:1–5. doi: 10.1016/j.autrev.2011.06.006. PMID: 21741498

[13] Man A Shojania K Phoon C Pal J de Badyn MH Pi D . An evaluation of autoimmune antibody testing patterns in a Canadian health region and an evaluation of a laboratory algorithm aimed at reducing unnecessary testing. Clin Rheumatol. (2013) 32:601–8. doi: 10.1007/s10067-012-2141-y. PMID: 23292519

[14] McLeroy KR Northridge ME Balcazar H Greenberg MR Landers SJ . Reporting guidelines and the American Journal of Public Health’s adoption of Preferred Reporting Items for Systematic Reviews and Meta-Analyses. Am J Public Health. (2012) 102:780–4. doi: 10.2105/AJPH.2011.300630. PMID: 22420806 PMC3483925

[15] Munn Z Moola S Lisy K Riitano D Tufanaru C . Methodological guidance for systematic reviews of observational epidemiological studies reporting prevalence and cumulative incidence data. Int J Evid Based Healthc. (2015) 13:147–53. doi: 10.1097/XEB.0000000000000054. PMID: 26317388

[16] Barendregt JJ Doi SA Lee YY Norman RE Vos T . Meta-analysis of prevalence. J Epidemiol Community Health. (2013) 67:974–8. doi: 10.1136/jech-2013-203104. PMID: 23963506

[17] DerSimonian R Laird N . Meta-analysis in clinical trials revisited. Contemp Clin Trials. (2015) 45:139–45. doi: 10.1016/j.cct.2015.09.002. PMID: 26343745 PMC4639420

[18] Borenstein M Hedges LV Higgins JP Rothstein HR . A basic introduction to fixed-effect and random-effects models for meta-analysis. Res Synth Methods. (2010) 1:97–111. doi: 10.1002/jrsm.12. PMID: 26061376

[19] Au EY Ip WK Lau CS Chan YT . Evaluation of a multiplex flow immunoassay versus conventional assays in detecting autoantibodies in systemic lupus erythematosus. Hong Kong Med J. (2018) 24:261–9. doi: 10.12809/hkmj177007. PMID: 29807953

[20] Blomberg S Ronnblom L Wallgren AC Nilsson B Karlsson-Parra A . Anti-SSA/Ro antibody determination by enzyme-linked immunosorbent assay as a supplement to standard immunofluorescence in antinuclear antibody screening. Scand J Immunol. (2000) 51:612–7. doi: 10.1046/j.1365-3083.2000.00735.x. PMID: 10849373

[21] Bossuyt X Luyckx A . Antibodies to extractable nuclear antigens in antinuclear antibody-negative samples. Clin Chem. (2005) 51:2426–7. doi: 10.1373/clinchem.2005.058552. PMID: 16306121

[22] Chandratilleke D Silvestrini R Culican S Campbell D Byth-Wilson K Swaminathan S . Comparison of two extractable nuclear antigen testing algorithms: ALBIA versus ELISA/line immunoassay. Pathology. (2016) 48:491–7. doi: 10.1016/j.pathol.2016.04.004. PMID: 27316331

[23] Dahle C Skogh T Aberg AK Jalal A Olcén P . Methods of choice for diagnostic antinuclear antibody (ANA) screening: benefit of adding antigen-specific assays to immunofluorescence microscopy. J Autoimmun. (2004) 22:241–8. doi: 10.1016/j.jaut.2003.12.004. PMID: 15041045

[24] Davis LA Goldstein B Tran V Keniston A Yazdany J Hirsh J . Applying Choosing Wisely: antinuclear antibody (ANA) and sub-serology testing in a safety net hospital system. Open Rheumatol J. (2015) 9:82–7. doi: 10.2174/1874312901409010082. PMID: 26862352 PMC4740967

[25] Desplat-Jego S Bardin N Larida B Sanmarco M . Evaluation of the BioPlex 2200 ANA screen for the detection of antinuclear antibodies and comparison with conventional methods. Ann N Y Acad Sci. (2007) 1109:245–55. doi: 10.1196/annals.1398.030. PMID: 17785313

[26] Ethington E Melrose E Stratman EJ . The relative timing, outcomes, and economic impact of antinuclear antibody (ANA) and extractable nuclear antigen (ENA) laboratory ordering. Clin Med Res. (2024) 22:123–6. doi: 10.3121/cmr.2024.1937. PMID: 39438147 PMC11495665

[27] Hayashi N Kawamoto T Mukai M Morinobu A Koshiba M Kondo S . Detection of antinuclear antibodies by use of an enzyme immunoassay with nuclear HEp-2 cell extract and recombinant antigens: comparison with immunofluorescence assay in 307 patients. Clin Chem. (2001) 47:1649–59. doi: 10.1093/clinchem/47.9.1649 11514399

[28] Hoffman IE Peene I Veys EM De Keyser F . Detection of specific antinuclear reactivities in patients with negative antinuclear antibody immunofluorescence screening tests. Clin Chem. (2002) 48:2171–6. doi: 10.1093/clinchem/48.12.2171 12446473

[29] Infantino M Manfredi M Grossi V Benucci M Morozzi G Tonutti E . An effective algorithm for the serological diagnosis of idiopathic inflammatory myopathies: the key role of anti-Ro52 antibodies. Clin Chim Acta. (2017) 475:15–9. doi: 10.1016/j.cca.2017.10.002. PMID: 28986052

[30] Infantino M Palterer B Biagiotti R Almerigogna F Benucci M Damiani A . Reflex testing of speckled cytoplasmic patterns observed in routine ANA HEp-2 indirect immunofluorescence with a multiplex anti-synthetase dot-blot assay: a multicentric pilot study. Immunol Res. (2018) 66:74–8. doi: 10.1007/s12026-017-8974-3. PMID: 29159696

[31] Jang J Kim S Kim HS Lee KA Park J Park Y . Comparison of antinuclear antibody profiles obtained using line immunoassay and fluorescence enzyme immunoassay. J Int Med Res. (2021) 49:3000605211014390. doi: 10.1177/03000605211014390. PMID: 34154430 PMC8236799

[32] Jaskowski TD Schroder C Martins TB Mouritsen CL Litwin CM Hill HR . Screening for antinuclear antibodies by enzyme immunoassay. Am J Clin Pathol. (1996) 105:468–73. doi: 10.1093/ajcp/105.4.468. PMID: 8604689

[33] Kern P Kron M Hiesche K . Measurement of antinuclear antibodies: assessment of different test systems. Clin Diagn Lab Immunol. (2000) 7:72–8. doi: 10.1128/CDLI.7.1.72-78.2000. PMID: 10618281 PMC95826

[34] Kim JM Ihm CH Sin DH Ihm MK Sim SC . Detection of anti-ENA and anti-dsDNA antibodies using line immunoassay in systemic autoimmune diseases. Korean J Lab Med. (2008) 28:353–61. doi: 10.3343/kjlm.2008.28.5.353. PMID: 18971616

[35] Lee SA Kahng J Kim Y Park YJ Han K Kwok SK . Comparative study of immunofluorescent antinuclear antibody test and line immunoassay detecting 15 specific autoantibodies in patients with systemic rheumatic disease. J Clin Lab Anal. (2012) 26:307–14. doi: 10.1002/jcla.21522. PMID: 22811366 PMC6807473

[36] Liu Q Xu H Guan X Shen Y Wen X Guo Y . Clinical significance of antinuclear and anti-extractable nuclear antigen antibody in childhood immune thrombocytopenia. Semin Thromb Hemost. (2017) 43:629–34. doi: 10.1055/s-0037-1599146. PMID: 28444667

[37] Nifli AP Notas G Mamoulaki M Niniraki M Ampartzaki V Theodoropoulos PA . Comparison of a multiplex, bead-based fluorescent assay and immunofluorescence methods for the detection of ANA and ANCA autoantibodies in human serum. J Immunol Methods. (2006) 311:189–97. doi: 10.1016/j.jim.2006.02.004. PMID: 16554066

[38] Pauling JD Salazar G Lu H Betteridge ZE Assassi S Mayes MD . Presence of anti-eukaryotic initiation factor-2B, anti-RuvBL1/2 and anti-synthetase antibodies in patients with antinuclear antibody-negative systemic sclerosis. Rheumatol (Oxford). (2018) 57:712–7. doi: 10.1093/rheumatology/kex458. PMID: 29294089

[39] Pottel H Wiik A Locht H Gordon T Roberts-Thomson P Abraham D . Clinical optimization and multicenter validation of antigen-specific cut-off values on the INNO-LIA ANA update for the detection of autoantibodies in connective tissue disorders. Clin Exp Rheumatol. (2004) 22:579–88. 15485011

[40] Robier C Amouzadeh-Ghadikolai O Stettin M Reicht G . Comparison of the clinical utility of the EliA CTD Screen to indirect immunofluorescence on HEp-2 cells. Clin Chem Lab Med. (2016) 54:1365–70. doi: 10.1515/cclm-2015-1051. PMID: 26677892

[41] Sebastian W Roy A Kini U Mullick S . Correlation of antinuclear antibody immunofluorescence patterns with immune profile using line immunoassay in the Indian scenario. Indian J Pathol Microbiol. (2010) 53:427–32. doi: 10.4103/0377-4929.68262. PMID: 20699497

[42] Sener AG Afsar I Demirci M . Evaluation of antinuclear antibodies by indirect immunofluorescence and line immunoassay methods: four years’ data from Turkey. APMIS. (2014) 122:1167–70. doi: 10.1111/apm.12275. PMID: 24735346

[43] Thomson KF Murphy A Goodfield MJ Misbah SA . Is it useful to test for antibodies to extractable nuclear antigens in the presence of a negative antinuclear antibody on HEp-2 cells? J Clin Pathol. (2001) 54:413. doi: 10.1136/jcp.54.5.413. PMID: 11328850 PMC1731413

[44] Vos PA Bast EJ Derksen RH . Cost-effective detection of non-anti-double-stranded DNA antinuclear antibody specificities in daily clinical practice. Rheumatol (Oxford). (2006) 45:629–35. doi: 10.1093/rheumatology/kei260. PMID: 16368728

[45] Yeo AL Ojaimi S Le S Leech M Morand E . Frequency and clinical utility of antibodies to extractable nuclear antigen in the setting of a negative antinuclear antibody test. Arthritis Care Res (Hoboken). (2023) 75:1595–601. doi: 10.1002/acr.24990. PMID: 35904968

[46] Yoon S Moon HW Kim H Hur M Yun YM . Clinical performance of two automated immunoassays, EliA CTD Screen and QUANTA Flash CTD Screen Plus, for antinuclear antibody screening. Ann Lab Med. (2022) 42:63–70. doi: 10.3343/alm.2022.42.1.63. PMID: 34374350 PMC8368234

[47] Pollock W Toh BH . Routine immunofluorescence detection of Ro/SS-A autoantibody using HEp-2 cells transfected with human 60 kDa Ro/SS-A. J Clin Pathol. (1999) 52:684–7. doi: 10.1136/jcp.52.9.684. PMID: 10655991 PMC501545

[48] Roberts-Thomson PJ Nikoloutsopoulos T Cox S Walker JG Gordon TP . Antinuclear antibody testing in a regional immunopathology laboratory. Immunol Cell Biol. (2003) 81:409–12. doi: 10.1046/j.1440-1711.2003.01181.x. PMID: 12969329

[49] Van der Pol P Bakker-Jonges LE Kuijpers JHSAM Schreurs MWJ . Analytical and clinical comparison of two fully automated immunoassay systems for the detection of autoantibodies to extractable nuclear antigens. Clin Chim Acta. (2018) 476:154–9. doi: 10.1016/j.cca.2017.11.014. PMID: 29170107

[50] Zafrir Y Gilburd B Carrasco MG Kivity S Sánchez-Castañón M López-Hoyos M . Evaluation of an automated chemiluminescent immunoassay kit for antinuclear antibodies in autoimmune diseases. Immunol Res. (2013) 56:451–6. doi: 10.1007/s12026-013-8416-9. PMID: 23579775

[51] Lee AYS Beroukas D Roberts-Thomson PJ . Utility of the HEp-2000 antinuclear antibody substrate. Ann Rheum Dis. (2020) 79:e67. doi: 10.1136/annrheumdis-2019-215519. PMID: 31088792

[52] Damoiseaux J Chan EK . Response to: “The utility of the HEp-2000 antinuclear antibody substrate” by Lee et al. Ann Rheum Dis. (2020) 79:e68. doi: 10.1136/annrheumdis-2019-215610. PMID: 31088789

