# Supplementary material for: Frequency of extractable nuclear antigen seropositivity among individuals seronegative for antinuclear antibodies on indirect immunofluorescence: a systematic review and meta-analysis
Source: Front Immunol. 2026 Apr 28;17:1745828. doi: 10.3389/fimmu.2026.1745828 (PMC13160898; doi:10.3389/fimmu.2026.1745828)
Supplement: Supplementary file 1 [file SupplementaryFile1.docx]

**Supplementary material**

1. **Detailed search strategies**

Overall, the search strategy was roughly identical across the following 4 databases: PubMed, EMBASE, Web Of Science and Scopus. The search for eligible studies was performed among all papers published prior to January 31, 2025. The following search string was used: (((Hep-2 negative) OR (negative ANA) OR (ANA-negative) OR (negative immunofluorescence)) AND ((ENA-positive) OR (positive ENA) OR (positive extractable nuclear antigen))) (Table S1). The literature search was carried out without any language restriction.

- 1. **Study search through PubMed**

PubMed database search was performed through the Advanced Search Builder. Three consecutive search strategies were applied for the Query Box: 1) Text Word, 2) MeSH Terms and 3) All Fields. All possible combinations of the following 2 categories of keywords were used for the extensive search:

1. Hep-2 negative OR negative ANA OR ANA-negative OR negative immunofluorescence
2. ENA-positive OR positive ENA OR positive extractable nuclear antigen
   1. **Study search through EMBASE**

EMBASE database search was carried out through the Quick search menu which automatically interprets and adjusts the search using both Emtree terms and keywords. The “Broad search” option was applied for the search box. Briefly, 2 search boxes were produced for the aforementioned 2 categories as separate concepts. Subsequently, all searches for each category were combined in order to obtain a final search without duplicates.

- 1. **Study search through Web Of Science**

Web Of Science database was made through its search engine by selecting the “All Fields” option. Keywords from the abovementioned 2 categories were added sequentially in order to refine the search results. Once all possible combinations had been applied, the search results were cross-tabulated through the “advanced Search” menu to eliminate duplicates.

- 1. **Study search through Scopus**

Scopus database search was conducted using the quick search engine. Briefly, 3 search boxes were produced in order to enter sequentially keywords from the aforementioned 2 categories. In order to refine search results the option “Article title, Abstract, Keywords” was selected for all search boxes. Subsequently, duplicates were eliminated by cross-tabulating search results.

**Search strategies across databases**

| **Search engine** | **Search strategies** |
| --- | --- |
| **PubMed** | ("HEp-2 negative" OR "Negative ANA" OR "ANA-negative" OR “negative immunofluorescence”)  AND  ("ENA-positive" OR "positive-ENA" OR " positive extractable nuclear antigen") |
| **Embase** | ('HEp-2 negative' OR 'Negative ANA' OR ‘ANA-negative’ OR 'negative immunofluorescence')  AND  ('ENA-positive' OR 'positive ENA' OR ‘positive extractable nuclear antigen’) |
| **Web of Science** | TS=("HEp-2 negative" OR "Negative ANA" OR "ANA-negative" OR “negative immunofluorescence”)  AND  TS=("ENA-positive" OR "positive-ENA" OR " positive extractable nuclear antigen") |
| **Scopus** | TITLE-ABS-KEY ("HEp-2 negative" OR "Negative ANA" OR "ANA-negative" OR “negative immunofluorescence)  AND TITLE-ABS-KEY ("ENA-positive" OR "positive-ENA" OR " positive extractable nuclear antigen") |

1. **Supplementary figures**

**
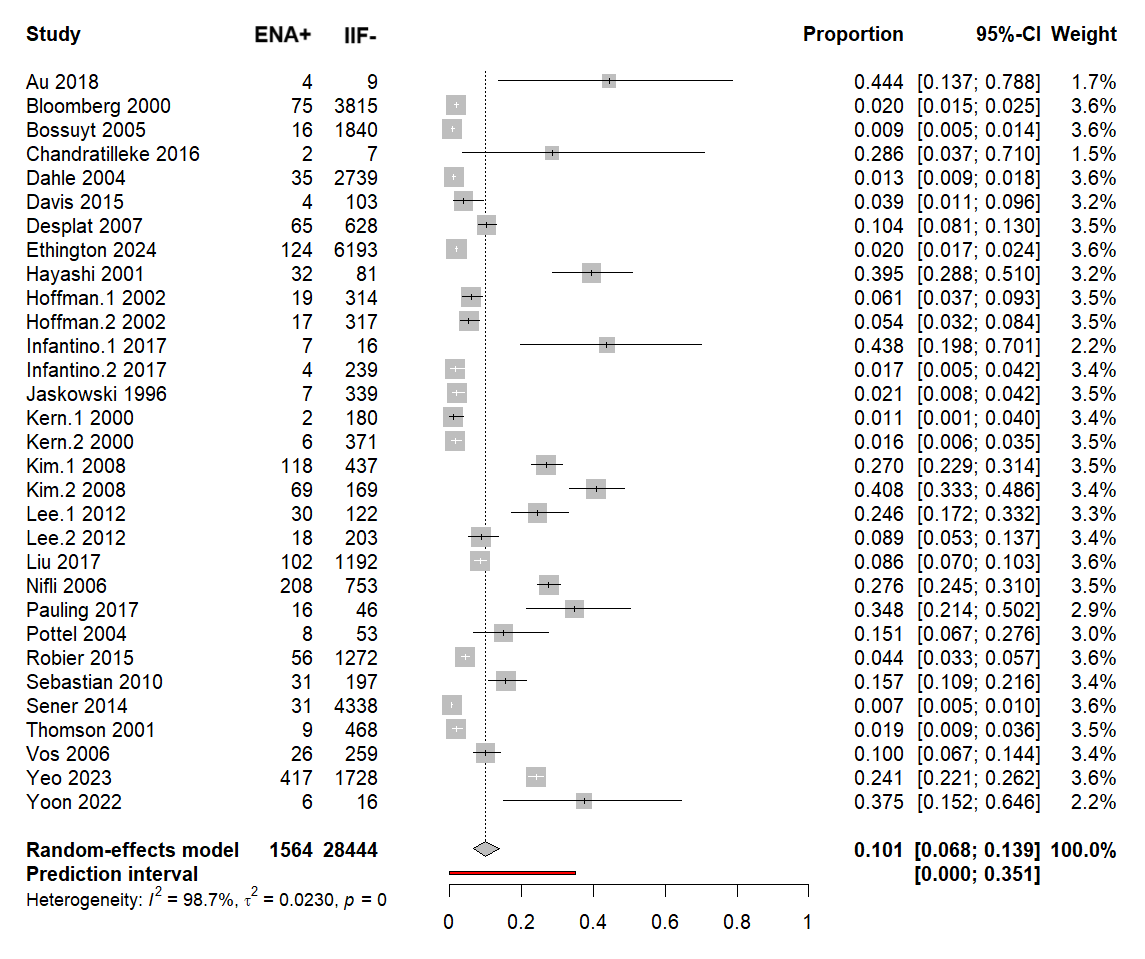
**

**Figure S1:** pooled proportion of ENA+/IIF- after removal of outlier studies (Jang.1 2021 and Jang.2 2021)


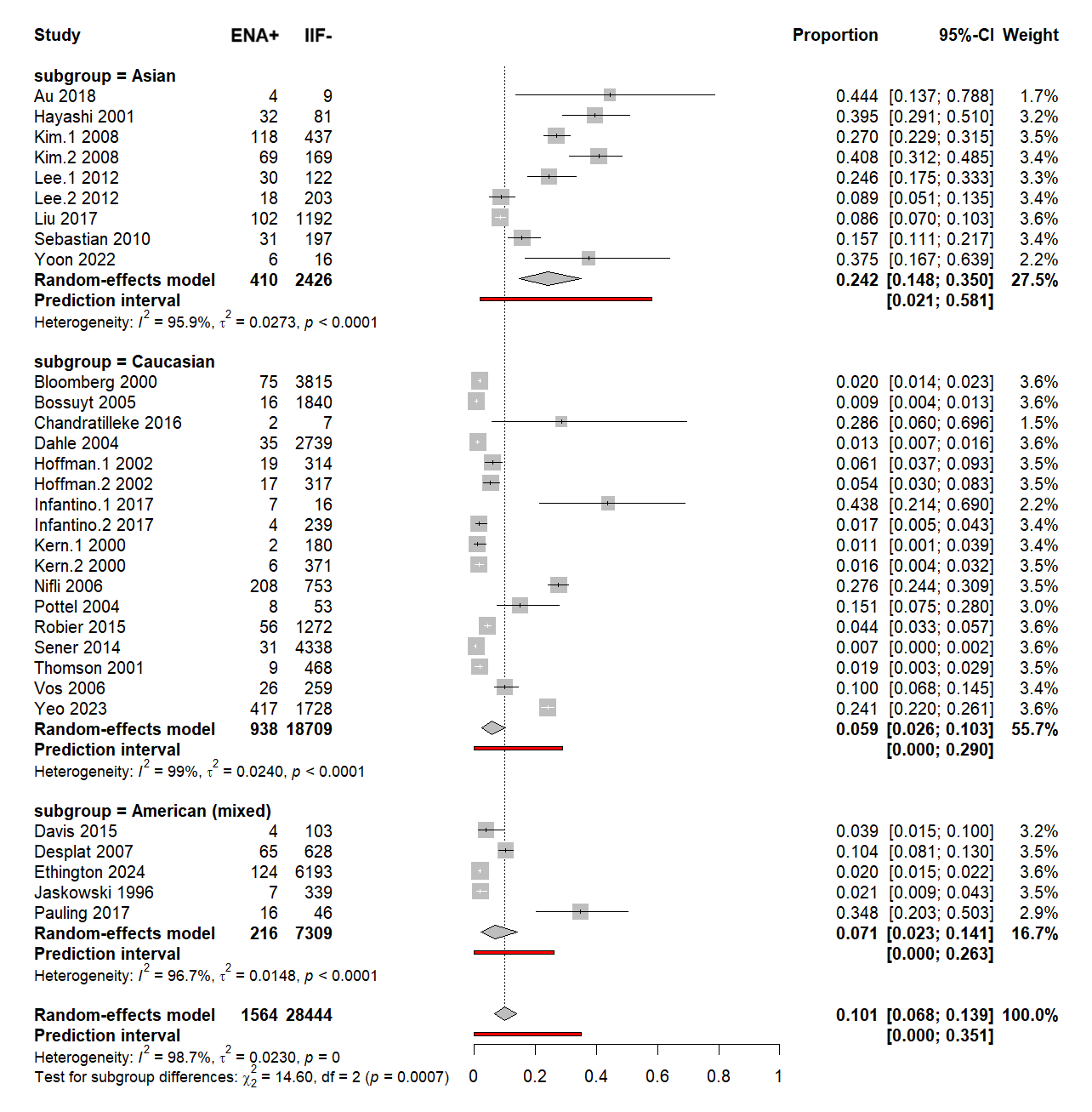


**Figure S2:** Subgroup analysis by ethnicity after removal of outlier studies (Jang.1 2021 and Jang.2 2021)


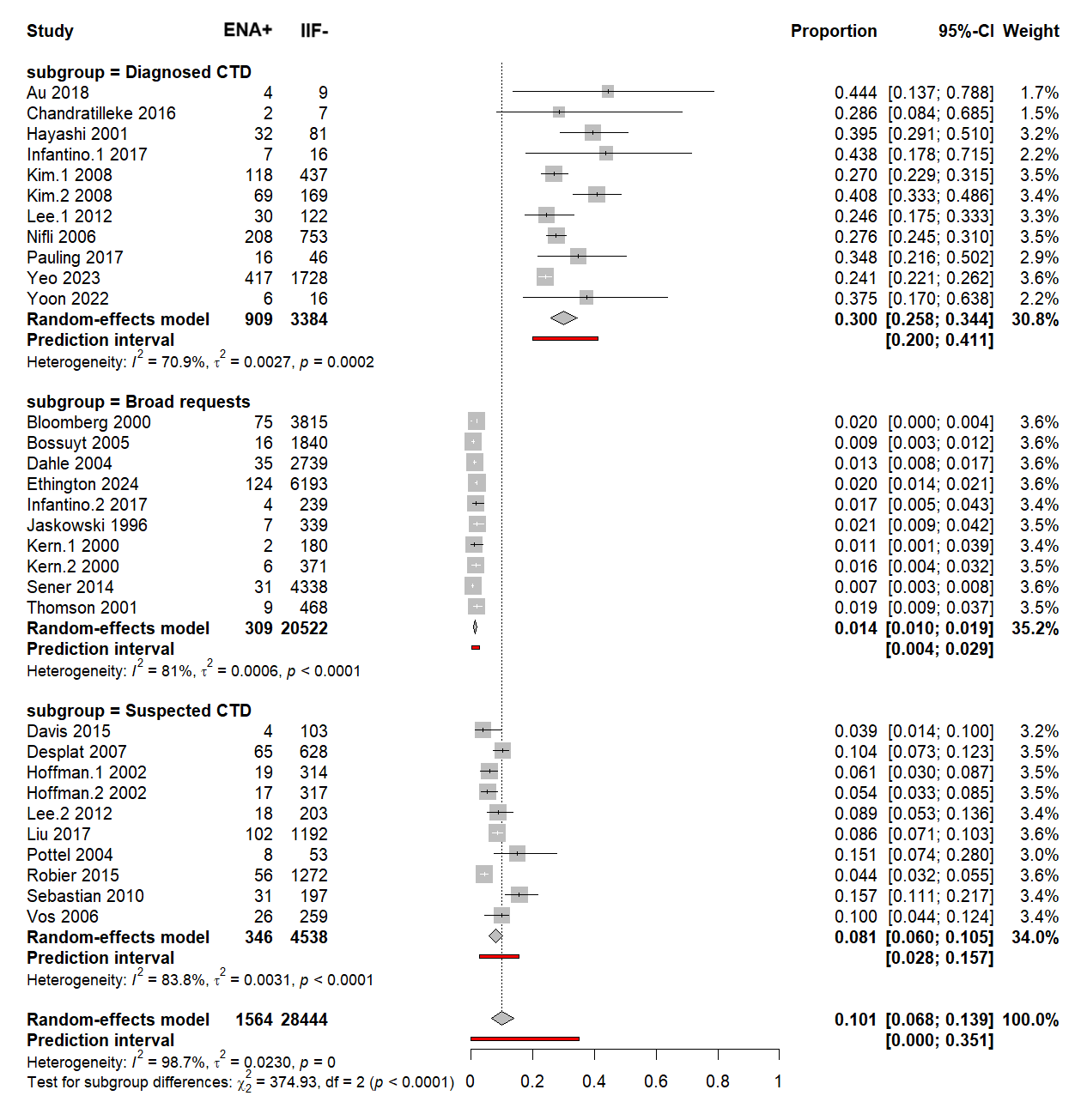


**Figure S3:** Subgroup analysis by context after removal of outlier studies (Jang.1 2021 and Jang.2 2021)


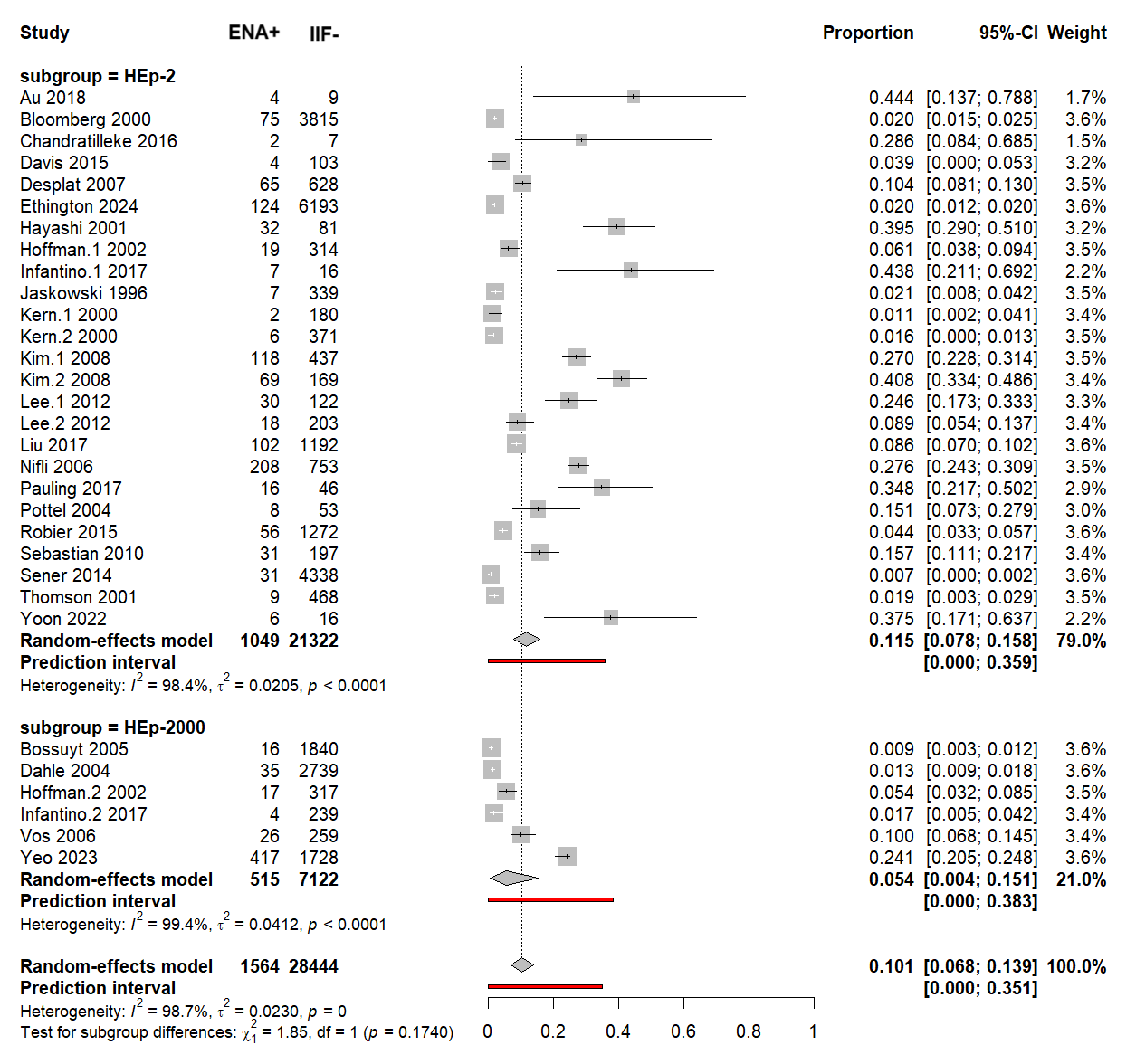


**Figure S4:** Subgroup analysis by ANA detection kit after removal of outlier studies (Jang.1 2021 and Jang.2 2021)


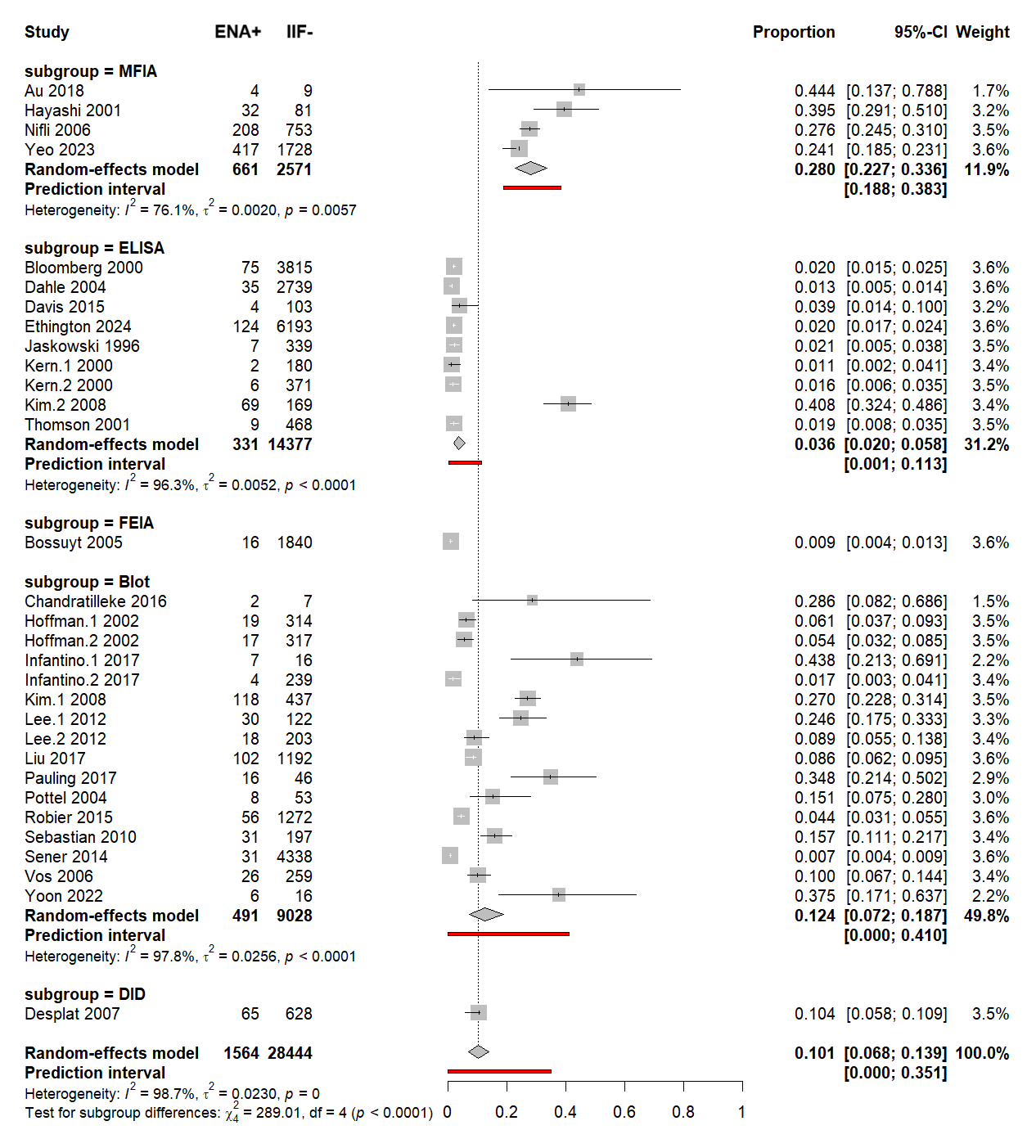


**Figure S5:** Subgroup analysis by ENA subtyping method after removal of outlier studies (Jang.1 2021 and Jang.2 2021)

1. **Supplementary Tables (S1-S7)**

**Supplementary Table S1**. PRISMA 2020 Checklist

| **Section and**  **Topic** | **Item**  **#** | **Checklist item** | **Location where item is reported** |
| --- | --- | --- | --- |
| **TITLE** | | |  |
| Title | 1 | Identify the report as a systematic review. | Page 1 |
| **ABSTRACT** | | |  |
| Abstract | 2 | See the PRISMA 2020 for Abstracts checklist. | Pages 2 and 3 |
| **INTRODUCTION** | | |  |
| Rationale | 3 | Describe the rationale for the review in the context of existing knowledge. | Pages 4, 5 and 6 |
| Objectives | 4 | Provide an explicit statement of the objective(s) or question(s) the review addresses. | Page 6 |
| **METHODS** | | |  |
| Eligibility criteria | 5 | Specify the inclusion and exclusion criteria for the review and how studies were grouped for the syntheses. | Page 7 |
| Information sources | 6 | Specify all databases, registers, websites, organisations, reference lists and other sources searched or consulted to identify studies. Specify the date when each source was last searched or consulted. | Pages 6 and 7 |
| Search strategy | 7 | Present the full search strategies for all databases, registers and websites, including any filters and limits used. | Pages 6 and 7, supplementary file 1 |
| Selection process | 8 | Specify the methods used to decide whether a study met the inclusion criteria of the review, including how many reviewers screened each record and each report retrieved, whether they worked independently, and if applicable, details of automation tools used in the process. | Page 7 |
| Data collection process | 9 | Specify the methods used to collect data from reports, including how many reviewers collected data from each report, whether they worked independently, any processes for obtaining or confirming data from study investigators, and if applicable, details of automation tools used in the process. | Pages 7 and 8 |
| Data items | 10a | List and define all outcomes for which data were sought. Specify whether all results that were compatible with each outcome domain in each study were sought (e.g. for all measures, time points, analyses), and if not, the methods used to decide which results to col lect. | Page 8 |
|  | 10b | List and define all other variables for which data were sought (e.g. participant and intervention characteristics, funding sources). Describe any assumptions made about any missing or unclear information. | Page 8 |
| Study risk of bias assessment | 11 | Specify the methods used to assess risk of bias in the included studies, including details of the tool(s) used, how many reviewers assessed each study and whether they worked independently, and if applicable, details of automation tools used in the process. | Page 8 |
| Effect measures | 12 | Specify for each outcome the effect measure(s) (e.g. risk ratio, mean difference) used in the synthesis or presentation of results. | Page 9 |
| Synthesis methods | 13a | Describe the processes used to decide which studies were eligible for each synthesis (e.g. tabulating the study intervention characteristics and comparing against the planned groups for each synthesis (item #5)). | Page 9 |
|  | 13b | Describe any methods required to prepare the data for presentation or synthesis, such as handling of missing summary statistics, or data conversions. | Page 9 |
|  | 13c | Describe any methods used to tabulate or visually display results of individual studies and syntheses. | Page 9 |
|  | 13d | Describe any methods used to synthesize results and provide a rationale for the choice(s). If meta-analysis was performed, describe the model(s), method(s) to identify the presence and extent of statistical heterogeneity, and software package(s) used. | Page 9 |
|  | 13e | Describe any methods used to explore possible causes of heterogeneity among study results (e.g. subgroup analysis, meta-regression). | Page 9 |
|  | 13f | Describe any sensitivity analyses conducted to assess robustness of the synthesized results. | Page 9 |
| Reporting bias assessment | 14 | Describe any methods used to assess risk of bias due to missing results in a synthesis (arising from reporting biases). | Page 10 |
| Certainty assessment | 15 | Describe any methods used to assess certainty (or confidence) in the body of evidence for an outcome. | Page 9 |

| **RESULTS** | | |  |
| --- | --- | --- | --- |
| Study selection | 16a | Describe the results of the search and selection process, from the number of records identified in the search to the number of studies included in the review, ideally using a flow diagram. | Page 10, Figure 1 |
|  | 16b | Cite studies that might appear to meet the inclusion criteria, but which were excluded, and explain why they were excluded. | Figure 1 |
| Study characteristics | 17 | Cite each included study and present its characteristics. | Page 10, Tables 1, 2 and 3 |
| Risk of bias in studies | 18 | Present assessments of risk of bias for each included study. | Table 1, Figure 2 |
| Results of individual studies | 19 | For all outcomes, present, for each study: (a) summary statistics for each group (where appropriate) and (b) an effect estimate and its precision  (e.g. confidence/credible interval), ideally using structured tables or plots. | Pages 10, 11, 12 and 13 |
| Results of syntheses | 20a | For each synthesis, briefly summarise the characteristics and risk of bias among contributing studies. | Pages 10, 11, 12 and 13 |
|  | 20b | Present results of all statistical syntheses conducted. If meta-analysis was done, present for each the summary estimate and its precision (e.g. confidence/credible interval) and measures of statistical heterogeneity. If comparing groups, describe the direction of the effect. | Pages 10, 11, 12 and 13 |
|  | 20c | Present results of all investigations of possible causes of heterogeneity among study results. | Pages 10, 11, 12 and 13 |
|  | 20d | Present results of all sensitivity analyses conducted to assess the robustness of the synthesized results. | Page 13, Figure 11 |
| Reporting biases | 21 | Present assessments of risk of bias due to missing results (arising from reporting biases) for each synthesis assessed. | NA |
| Certainty of evidence | 22 | Present assessments of certainty (or confidence) in the body of evidence for each outcome assessed. | Pages 10, 11, 12 and 13 |
| **DISCUSSION** | | |  |
| Discussion | 23a | Provide a general interpretation of the results in the context of other evidence. | Pages 13, 14, 15, 16 and 17 |
|  | 23b | Discuss any limitations of the evidence included in the review. | Page 17 |
|  | 23c | Discuss any limitations of the review processes used. | Page 17 |
|  | 23d | Discuss implications of the results for practice, policy, and future research. | Pages 16, 17 |
| **OTHER INFORMATION** | | |  |
| Registration and protocol | 24a | Provide registration information for the review, including register name and registration number, or state that the review was not registered. | Pages 3 and 10 |
|  | 24b | Indicate where the review protocol can be accessed, or state that a protocol was not prepared. | Pages 3 and 10 |
|  | 24c | Describe and explain any amendments to information provided at registration or in the protocol. | NA |
| Support | 25 | Describe sources of financial or non-financial support for the review, and the role of the funders or sponsors in the review. | Page 18 |
| Competing interests | 26 | Declare any competing interests of review authors. | Page 18 |
| Availability of data, code and other materials | 27 | Report which of the following are publicly available and where they can be found: template data collection forms; data extracted from included studies; data used for all analyses; analytic code; any other materials used in the review. | Page 18 |

*From:* Page MJ, McKenzie JE, Bossuyt PM, Boutron I, Hoffmann TC, Mulrow CD, et al. The PRISMA 2020 statement: an updated guideline for reporting systematic reviews. BMJ 2021;372:n71. doi:10.1136/bmj.n71

**Supplementary Table S2**.
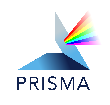
 **PRISMA 2020 for Abstracts Checklist**

| **Section and Topic** | **Item #** | **Checklist item** | **Reported (Yes/No)** |
| --- | --- | --- | --- |
| **TITLE** | | |  |
| Title | 1 | Identify the report as a systematic review. | Yes |
| **BACKGROUND** | | |  |
| Objectives | 2 | Provide an explicit statement of the main objective(s) or question(s) the review addresses. | Yes |
| **METHODS** | | |  |
| Eligibility criteria | 3 | Specify the inclusion and exclusion criteria for the review. | Yes |
| Information sources | 4 | Specify the information sources (e.g. databases, registers) used to identify studies and the date when each was last searched. | Yes |
| Risk of bias | 5 | Specify the methods used to assess risk of bias in the included studies. | Yes |
| Synthesis of results | 6 | Specify the methods used to present and synthesise results. | Yes |
| **RESULTS** | | |  |
| Included studies | 7 | Give the total number of included studies and participants and summarise relevant characteristics of studies. | Yes |
| Synthesis of results | 8 | Present results for main outcomes, preferably indicating the number of included studies and participants for each. If meta-analysis was done, report the summary estimate and confidence/credible interval. If comparing groups, indicate the direction of the effect (i.e. which group is favoured). | Yes |
| **DISCUSSION** | | |  |
| Limitations of evidence | 9 | Provide a brief summary of the limitations of the evidence included in the review (e.g. study risk of bias, inconsistency and imprecision). | NA |
| Interpretation | 10 | Provide a general interpretation of the results and important implications. | Yes |
| **OTHER** | | |  |
| Funding | 11 | Specify the primary source of funding for the review. | NA |
| Registration | 12 | Provide the register name and registration number. | Yes |

*From:*  Page MJ, McKenzie JE, Bossuyt PM, Boutron I, Hoffmann TC, Mulrow CD, et al. The PRISMA 2020 statement: an updated guideline for reporting systematic reviews. BMJ 2021;372:n71. doi: 10.1136/bmj.n71. This work is licensed under CC BY 4.0. To view a copy of this license, visit <https://creativecommons.org/licenses/by/4.0/>

**Supplementary Table S3**: Study characteristics

| **Ref** | **Study** | **Departments** | **Timeline of data collection** | **Community**  **/Hospital** | **Country** | **Ethnicity** | **Age** | **Gender-ratio**  **(M/F)** | **Reason for ANA / ENA request** | **CTD** | **JBI**  **score⁂** |
| --- | --- | --- | --- | --- | --- | --- | --- | --- | --- | --- | --- |
| **[19]** | Au 2018 | Lab(immunology)/Rheumatology | December 2013 | Hospital | Hong Kong | Asian | 46.8 | 0.09375 | Diagnosed CTD‡ | SLE⸸ | 9 |
| **[20]** | Bloomberg 2000 | Lab (general)/Rheumatology | 12 months | Hospital | Sweden | Caucasian | NS† | NS | Broad requests | NS | 8 |
| **[21]** | Bossuyt 2005 | Lab (immunology) | 15 weeks | Hospital | Belgium | Caucasian | NS | NS | Broad requests | NS | 8 |
| **[22]** | Chandratilleke 2016 | Lab (immunology) | June-July 2015 | Hospital | Australia | Caucasian | NS | NS | Diagnosed CTD | Mixed⸶ | 8 |
| **[23]** | Dahle 2004 | Lab(immunology)/Rheumatology | 12 months | Hospital | Sweden | Caucasian | 51.5 | 0.42 | Broad requests | NS | 9 |
| **[24]** | Davis 2015 | Lab (general) | 2005 to 2011 | Hospital | USA | American* | 45.2 | 0.4687 | Suspected CTD | NS | 9 |
| **[25]** | Desplat 2007 | Lab (immunology) | 3 weeks | Hospital | USA | American | NS | NS | Suspected CTD | NS | 9 |
| **[26]** | Ethington 2024 | Dermatology | 2002 to 2020 | Hospital | USA | American | 48.7 | 0.497 | Broad requests | NS | 9 |
| **[27]** | Hayashi 2001 | Lab (general) | NS | Hospital | Japan | Asian | NS | NS | Diagnosed CTD | Mixed | 8 |
| **[28]** | Hoffman.1 2002 | Rheumatology | 1996 to 1999 | Hospital | Belgium | Caucasian | 44.9 | 0.6 | Suspected CTD | NS | 9 |
| **[28]** | Hoffman.2 2002 | Rheumatology | 1996 to 1999 | Hospital | Belgium | Caucasian | 44.9 | 0.6 | Suspected CTD | NS | 9 |
| **[29]** | Infantino.1 2017 | Lab(immunology)/Rheumatology | 18 months | Hospital | Italy | Caucasian | 57 | 0.214 | Diagnosed CTD | IIM⸷ | 9 |
| **[30]** | Infantino.2 2017 | Lab(immunology)/medicine | NS | Hospital | Italy | Caucasian | NS | NS | Broad requests | _ | 8 |
| **[31]** | Jang.1 2021 | Lab (general) | NS | Hospital | South Korea | Asian | 55 | 0.31 | Diagnosed CTD | Mixed | 9 |
| **[31]** | Jang.2 2021 | Lab (general) | NS | Hospital | South Korea | Asian | 55 | 0.31 | Diagnosed CTD | Mixed | 9 |
| **[32]** | Jaskowski 1996 | Lab (general) | NS | Hospital | USA | American | NS | NS | Broad requests | NS | 8 |
| **[33]** | Kern.1 2000 | Lab (immunology) | NS | Hospital | Germany | Caucasian | 44.35 | 0.697 | Broad requests | NS | 9 |
| **[33]** | Kern.2 2000 | Lab (immunology) | NS | Hospital | Germany | Caucasian | 44.35 | 0.697 | Broad requests | NS | 9 |
| **[34]** | Kim.1 2008 | Lab (general)/Rheumatology | 2005 to 2006 | Hospital | South Korea | Asian | 43.8 | 0.21 | Diagnosed CTD | Mixed | 9 |
| **[34]** | Kim.2 2008 | Lab (general)/Rheumatology | 2005 to 2006 | Hospital | South Korea | Asian | 43.8 | 0.21 | Diagnosed CTD | Mixed | 9 |
| **[35]** | Lee.1 2012 | Lab(immunology)/Rheumatology | NS | Hospital | South Korea | Asian | NS | NS | Diagnosed CTD | Mixed | 8 |
| **[35]** | Lee.2 2012 | Lab(immunology)/Rheumatology | NS | Hospital | South Korea | Asian | NS | NS | Suspected CTD | NS | 8 |
| **[36]** | Liu 2017 | Lab(general)/hematology | 2010 to 2014 | Hospital | China | Asian | 3.8 | 1.375 | Suspected CTD | NS | 9 |
| **[37]** | Nifli 2006 | Lab(immunology)/medicine | 2004 to 2005 | Hospital | Greece | Caucasian | NS | NS | Diagnosed CTD | Mixed | 8 |
| **[38]** | Pauling 2017 | Lab(immunology)/Rheumatology | NS | Hospital | USA | American | NS | NS | Diagnosed CTD | SSc⁑ | 8 |
| **[39]** | Pottel 2004 | Lab (immunology) | NS | Hospital | Denmark | Caucasian | NS | NS | Suspected CTD | NS | 8 |
| **[40]** | Robier 2015 | Lab(general)/Internal medicine | 2014 to 2015 | Hospital | Austria | Caucasian | 56 | 0.44 | Suspected CTD | NS | 9 |
| **[41]** | Sebastian 2010 | Lab(general)/medicine | 27 months | Hospital | India | Asian | NS | NS | Suspected CTD | NS | 8 |
| **[42]** | Sener 2014 | Lab (microbiology) | 2009 - 2013 | Hospital | Turkey | Caucasian | NS | NS | Broad requests | NS | 8 |
| **[43]** | Thomson 2001 | Dermatology | 12 months | Hospital | UK | Caucasian | NS | NS | Broad requests | NS | 8 |
| **[44]** | Vos 2006 | Lab(immunology)/Rheumatology | 2003 to 2004 | Hospital | Netherlands | Caucasian | 44 | 0.46 | Suspected CTD | NS | 9 |
| **[45]** | Yeo 2023 | Lab (immunology) | 2011 to 2018 | Hospital | Australia | Caucasian | 56.1 | 0.6 | Diagnosed CTD | Mixed | 9 |
| **[46]** | Yoon 2022 | Lab (general) | 2018 to 2019 | Hospital | Korea | Asian | 48 | 0.19 | Diagnosed CTD | Mixed | 9 |

*American: mixed: Caucasians + Afro-Americans + Amerindians; †NS: Not Specified; ‡CTD: connective tissue diseases; ⸸SLE: Systemic lupus erythematosus; ⸶Mixed: systemic lupus erythematosus + Sjögren syndrome, systemic sclerosis, mixed connective tissue disease and idiopathic inflammatory myopathies; ⸷IIM: idiopathic inflammatory myopathies ⁑SSc: systemic sclerosis; ⁂JBI critical appraisal for systematic reviews of prevalence and incidence studies

*American: mixed: Caucasians + Afro-Americans + Amerindians; †NS: Not Specified; ‡CTD: connective tissue diseases; ⸸SLE: Systemic lupus erythematosus; ⸶Mixed: systemic lupus erythematosus + Sjögren syndrome, systemic sclerosis, mixed connective tissue disease and idiopathic inflammatory myopathies; ⸷IIM: idiopathic inflammatory myopathies ⁑SSc: systemic sclerosis; ⁂JBI critical appraisal for systematic reviews of prevalence and incidence studies

**Supplementary Table S4**: ANA and ENA detection details

| **Study** | **ANA detection Kit** | **Conjugate** | **IIF cut-off** | **Microscope** | **ENA method** | **ENA+** | **ANA-IIF-** |
| --- | --- | --- | --- | --- | --- | --- | --- |
| Au 2018 | Kallestad (HEp-2), Bio-Rad, Hercules | Anti-IgG | 80 | NS | MFIA* | 4 | 9 |
| Bloomberg 2000 | NS (HEp-2) | NS | 100 | NS | ELISA† | 75 | 3815 |
| Bossuyt 2005 | Immunoconcepts (HEp-2000) | Anti-IgG | 80 | Mercury light | FEIA‡ | 16 | 1840 |
| Chandratilleke 2016 | Euroimmun (HEp-20-10) | Anti-IgG | 80 | NS | Dot-blot | 2 | 7 |
| Dahle 2004 | Immunoconcepts (HEp-2000) | Anti-IgG | 100 | Mercury light | ELISA | 35 | 2739 |
| Davis 2015 | NS (HEp-2) | Anti-IgG | 40 | NS | ELISA | 4 | 103 |
| Desplat 2007 | Bio-Rad (HEp-2) | Anti-GAM | 100 | NS | DID⸸ | 65 | 628 |
| Ethington 2024 | NS (HEp-2) | NS | 80 | NS | ELISA | 124 | 6193 |
| Hayashi 2001 | Diatron (HEp-2) | Anti-GAM | 80 | NS | MFIA | 32 | 81 |
| Hoffman.1 2002 | MeDiCa (HEp-2) | Anti-IgG | 40 | Mercury light | Dot-blot | 19 | 314 |
| Hoffman.2 2002 | Immunoconcepts (HEp-2000) | Anti-IgG | 40 | Mercury light | Dot-blot | 17 | 317 |
| Infantino.1 2017 | Euroimmun (HEp-20-10) | Anti-IgG | 80 | NS | Dot-blot | 7 | 16 |
| Infantino.2 2017 | Immunoconcepts (HEp-2000) | Anti-IgG | 80 | NS | Dot-blot | 4 | 239 |
| Jang.1 2021 | INOVA (HEp-2) | Anti-IgG | 80 | NS | Dot-blot | 48 | 54 |
| Jang.2 2021 | INOVA (HEp-2) | Anti-IgG | 80 | NS | FEIA | 53 | 54 |
| Jaskowski 1996 | INOVA (HEp-2) | Anti-IgG | 40 | Mercury light | ELISA | 7 | 339 |
| Kern.1 2000 | Rat liver + HEp-2 (Kallestad / Sanofi) | NS | 40 | NS | ELISA | 2 | 180 |
| Kern.2 2000 | Rat liver + HEp-2 (Kallestad / Sanofi) | NS | 40 | NS | ELISA | 6 | 371 |
| Kim.1 2008 | Bio-Rad (HEp-2) | Anti-GAM | 160 | NS | Dot-blot | 118 | 437 |
| Kim.2 2008 | Bio-Rad (HEp-2) | Anti-GAM | 160 | NS | ELISA | 69 | 169 |
| Lee.1 2012 | Euroimmun (HEp-20-10/liver) | Anti-IgG | 100 | NS | Dot-blot | 30 | 122 |
| Lee.2 2012 | Euroimmun (HEp-20-10/liver) | Anti-IgG | 100 | NS | Dot-blot | 18 | 203 |
| Liu 2017 | Euroimmun (HEp-20-10) | Anti-IgG | 100 | NS | Dot-blot | 102 | 1192 |
| Nifli 2006 | INOVA (HEp-2) | Anti-IgG | 160 | NS | MFIA | 208 | 753 |
| Pauling 2017 | INOVA (HEp-2) | Anti-IgG | 40 | NS | Immunoblot | 16 | 46 |
| Pottel 2004 | NS (HEp-2) | Anti-IgG | 80 | NS | Dot-blot | 8 | 53 |
| Robier 2015 | Orgentec (HEp-2 | Anti-IgG | 160 | NS | Dot-blot | 56 | 1272 |
| Sebastian 2010 | Euroimmun (HEp-20-10/liver) | Anti-IgG | 100 | NS | Dot-blot | 31 | 197 |
| Sener 2014 | Euroimmun (HEp-20-10/liver) | Anti-IgG | 100 | NS | Dot-blot | 31 | 4338 |
| Thomson 2001 | NS (HEp-2) | NS | NS | NS | ELISA | 9 | 468 |
| Vos 2006 | Immunoconcepts (HEp-2000) | Anti-IgG | 40 | NS | Dot-blot | 26 | 259 |
| Yeo 2023 | Immunoconcepts (HEp-2000) | Anti-IgG | 160 | NS | MFIA | 417 | 1728 |
| Yoon 2022 | Kallestad (HEp-2) | Anti-IgG | 80 | NS | Dot-blot | 6 | 16 |

* MFIA: Multiplex flow immunoassay; †ELISA: Enzyme-Linked ImmunoSorbent Assay; ‡ FEIA: fluorescent enzyme immunoassay; ⸸Ouchterlony double immunodiffusion

**Supplementary Table S5.** Frequencies of anti-dsDNA and individual ENA Abs in patients with negative ANA by IIF

| **Study** | **Anti-dsDNA** | **Individual ENA Ab frequencies** | | | | | | | | |
| --- | --- | --- | --- | --- | --- | --- | --- | --- | --- | --- |
|  |  | **Histone** | **SSA/Ro** | **SSB/La** | **Sm** | **RNP** | **CENP-B** | **Scl-70** | **Ribosome** | **ARS** |
| Au 2018 | NS* | NS | 0,429 | 0,264 | 0,243 | 0,393 | NS | 0,05 | NS | 0,07 |
| Bloomberg 2000 | NS | NS | 0,019659 | NS | NS | NS | NS | NS | NS | NS |
| Bossuyt 2005 | NS | 0 | 0,005978 | 0 | 0,001087 | 0,001087 | 0 | 0,000543 | 0 | 0,000543 |
| Chandratilleke 2016 | NS | 0 | 0 | 0 | 0 | 0,142857 | 0 | 0 | 0 | 0,142857 |
| Dahle 2004 | NS | NS | 0,011628 | 0,006939 | 0,005482 | 0,005111 | 0,004741 | 0,002185 | NS | 0,000371 |
| Davis 2015 | 0,0096 | NS | 0,0192 | 0 | 0 | 0,0096 | 0 | 0,01923 | NS | NS |
| Desplat 2007 | 0,035326 | 0,074275 | 0,09058 | 0,035326 | 0,1992 | 0,05344 | 0,01721 | 0,013587 | 0,009964 | 0,003623 |
| Ethington 2024 | 0,004685 | NS | 0,004365 | 0,002263 | 0,001622 | 0,006627 | 0,000481 | 0,001462 | NS | 0,000641 |
| Hayashi 2001 | 0 | NS | 0,21875 | 0,15625 | 0,09375 | 0,21875 | 0,0625 | 0,03125 | NS | 0,03125 |
| Hoffman.1 2002 | NS | 0,007564 | 0,026473 | 0,026473 | 0,003782 | 0,022691 | 0 | 0,003782 | 0 | 0,007564 |
| Hoffman.2 2002 | NS | 0,006703 | 0,023462 | 0,023462 | 0,003352 | 0,02011 | 0 | 0,003352 | 0 | 0,006703 |
| Infantino.1 2017 | 0 | 0 | 0,374938 | 0 | 0 | 0 | 0 | 0 | 0 | 0,249813 |
| Infantino.2 2017 | NS | NS | NS | NS | NS | NS | NS | NS | NS | 0,000279 |
| Jang.1 2021 | 0,173 | NS | 0,612 | 0,115 | 0,115 | 0,183 | 0,089 | 0,05 | 0 | 0,016 |
| Jang.2 2021 | 0,173 | NS | 0,612 | 0,115 | 0,115 | 0,183 | 0,089 | 0,05 | 0 | 0,016 |
| Jaskowski 1996 | 0,00885 | 0,00885 | 0,00295 | 0 | 0 | 0 | 0 | 0 | NS | 0 |
| Kern.1 2000 | NS | NS | NS | NS | NS | NS | NS | NS | NS | NS |
| Kern.2 2000 | NS | NS | NS | NS | NS | NS | NS | NS | NS | NS |
| Kim.1 2008 | 0 | 0 | 0,085821 | 0,022388 | 0 | 0,007463 | 0 | 0,00373 | 0 | 0,007463 |
| Kim.2 2008 | 0 | 0 | 0,136095 | 0,0355 | 0 | 0,011834 | 0 | 0,005917 | 0 | 0,011834 |
| Lee.1 2012 | 0,03042 | 0,035218 | 0,093842 | 0,03649 | 0,045936 | 0,040317 | 0,02039 | 0,006951 | 0 | 0,003244 |
| Lee.2 2012 | 0,00748 | 0,001277 | 0,021255 | 0,00821 | 0,002463 | 0,008393 | 0,00483 | 0,001642 | 0 | 0,00073 |
| Liu 2017 | 0,001677 | NS | 0,063758 | 0,006711 | 0,01174 | 0,01174 | 0,003354 | 0,016772 | 0 | NS |
| Nifli 2006 | NS | NS | NS | NS | NS | NS | NS | NS | NS | NS |
| Pauling 2017 | NS | NS | 0,047619 | NS | NS | NS | NS | NS | NS | 0,152174 |
| Pottel 2004 | NS | 0,021212 | 0,027273 | 0,00303 | 0,009091 | 0,051515 | 0,00303 | 0 | 0,00303 | 0,00303 |
| Robier 2015 | 0,00943 | 0 | 0,00864 | 0,000786 | 0,00157 | 0,001572 | 0 | 0 | 0 | 0,00157 |
| Sebastian 2010 | 0,005076 | 0,005076 | 0,13198 | 0 | 0 | 0 | 0 | 0,02538 | 0 | 0 |
| Sener 2014 | 0,000461 | 0 | 0,006224 | 0,000692 | 0 | 0 | 0 | 0,000461 | 0 | 0 |
| Thomson 2001 | NS | NS | 0,019231 | 0 | 0 | 0 | NS | NS | NS | NS |
| Vos 2006 | NS | 0,015439 | 0,030888 | 0,00772 | 0,027004 | 0,019304 | 0 | 0,011583 | 0 | 0,003861 |
| Yeo 2023 | NS | 0,019788 | 0,110766 | 0,008688 | 0,001689 | 0 | 0,001689 | 0,025097 | 0,002594 | 0,010377 |
| Yoon 2022 | NS | 0 | 0,140625 | 0 | 0 | 0 | 0 | 0 | 0,0625 | 0 |

*NS: not specified; dsDNA: double-stranded DNA, SSA: Sjögren Syndrome related antigen A, SSB: Sjögren Syndrome related antigen B, Sm: Smith, RNP: RiboNucleoProtein, CENP-B: major centromere autoantigen B, Scl-70: Scleroderma 70 Kd antigen, ARS: anti-tRNA synthetase Ab

**Supplementary Table S6**: Frequency of negative-ANA on IIF / positive-ENA and subgroup analyses

| **Subgroup** | **Proportion [95% CI]** | | **I^2^** | ***p*-value** | **Tau^2^** | **95% PI** |
| --- | --- | --- | --- | --- | --- | --- |
| **Overall (n=33)** | 14.1% [10% – 18.7%] | | 99% | < 0.0001 | 0.0282 | 0% – 44.2% |
| **Without outliers (n=31)** | 10.1% [6.8% – 13.9%] | | 99% | < 0.0001 | 0.0230 | 0% – 35.1% |
| **Ethnicity** |  |  |  |  |  |  |
| American (n=5) | 7.1% [2.3% – 14.1%] | | 97% | < 0.0001 | 0.0148 | 0% – 26.3% |
| Caucasian (n=17) | 5.9% [2.6% – 10.3%] | | 99% | < 0.0001 | 0.0240 | 0% – 29% |
| Asian (n=11) | 38.7% [23.3% – 55.3%] | | 98% | < 0.0001 | 0.0717 | 0.6% – 89.3% |
| Asian without outliers (n=9) | 24.2% [14.8% – 35%] | | 96% | < 0.0001 | 0.0273 | 2.1% – 58.1% |
| **Test for subgroup differences: *p* < 0.0001** | | | | | | |
| **Context** |  |  |  |  |  |  |
| Broad requests (n=10) | 1.4% [1% – 1.9%] | | 81% | < 0.0001 | 0.0006 | 0.4% – 2.9% |
| Suspected CTD (n=10) | 8.1% [6% – 10.5%] | | 84% | < 0.0001 | 0.0031 | 2.8% – 15.7% |
| Diagnosed CTD (n=13) | 44.1% [33.2% – 54.6%] | | 96% | < 0.0001 | 0.0286 | 12.6% – 78.3% |
| Diagnosed CTD without outliers (n=11) | 30% [25.8% – 34.4%] | | 70% | < 0.0001 | 0.0027 | 20% – 41.1% |
| **Test for subgroup differences: *p*** **< 0.0001** | | | | | | |
| **ANA detection method** |  |  |  |  |  |  |
| HEp-2000 (n=6) | 5.4% [0.4% – 15.1%] | | 99% | < 0.0001 | 0.0412 | 0% – 38.3% |
| HEp-2 (n=27) | 16.8% [12% – 22.3%] | | 99% | < 0.0001 | 0.0281 | 0.2% – 48% |
| HEp-2 without outliers (n=25) | 11.5% [7.8% – 15.8%] | | 98% | < 0.0001 | 0.0205 | 0% – 35.9% |
| **Test for subgroup differences: *p* = 0.0314** | | | | | | |
| **IIF cut-off** |  |  |  |  |  |  |
| 1/40 | 5.5% [2.6% - 9.4%] |  | 90% | < 0.0001 | 0.0097 | 0% – 18.6% |
| 1/80 or 1/100 | 14.8% [10.6% - 19.6%] |  | 98% | < 0.0001 | 0.0159 | 1.3% – 37.4% |
| 1/160 | 23.1% [11.3% - 37.6%] |  | 99% | < 0.0001 | 0.0320 | 1.3% – 60.1% |
| **Test for subgroup differences: *p* = 0.0005** | | | | | | |
| **ENA detection method** |  |  |  |  |  |  |
| DID (n=1) | 10.4% [8.2% – 13%] | | _ | _ | _ | _ |
| Dot-blot/Immunoblot (n=17) | 16.3% [9.8% – 23.9%] | | 98% | < 0.0001 | 0.0339 | 0% – 51.2% |
| ELISA (n=9) | 3.6% [2% – 5.8%] | | 96% | < 0.0001 | 0.0052 | 0.1% – 11.3% |
| FEIA (n=2) | 46.4% [0% – 100%] | | 100% | < 0.0001 | 0.8586 | _ |
| MFIA (n=4) | 28% [22.7% – 33.6%] | | 76% | < 0.0001 | 0.0057 | 18.8% – 38.3% |
| **Test for subgroup differences: *p* < 0.0001** | | | | | | |

**Supplementary Table S7**: Results of meta-regressions for the frequency of negative-ANA on IIF / positive-ENA

| **Covariate** | **Coefficients** | **Bounds** | | **Std. error** | ***p*-value** | |
| --- | --- | --- | --- | --- | --- | --- |
|  |  | **Lower** | **Upper** |  | **Univariable** | **Multivariable** |
| **Year of publication** | 0.016 | 0.004 | 0.028 | 0.006 | **0.012** | **0.004** |
| **Age** | 0.009 | -0.005 | 0.024 | 0.007 | 0.198 | 0.508 |
| **Gender-ratio (M/F)** | -0.625 | -1.162 | -0.087 | 0.274 | **0.023** | 0.626 |
| **Serum dilution cut-off** | 0.0015 | 0.0002 | 0.0029 | 0.0007 | **0.014** | **0.006** |
| **dsDNA Ab %** | 5.566 | 3.660 | 7.473 | 0.973 | **< 0.0001** | 0.562 |
| **Anti-histone Ab %** | 0.120 | -4.616 | 4.856 | 2.416 | 0.960 | 0.625 |
| **Anti-SSA/Ro Ab %** | 1.842 | 1.553 | 2.131 | 0.147 | **< 0.0001** | **< 0.0001** |
| **Anti-SSB/La Ab %** | 3.605 | 1.910 | 5.301 | 0.865 | **< 0.0001** | 0.191 |
| **Anti-Sm Ab %** | 2.591 | 0.828 | 4.354 | 0.899 | **0.004** | 0.112 |
| **Anti-RNP Ab%** | 2.407 | 1.296 | 3.517 | 0.567 | **< 0.0001** | 0.212 |
| **CENP-B Ab %** | 10.178 | 7.297 | 13.058 | 1.470 | **< 0.0001** | 0.095 |
| **Anti-Scl70 Ab %** | 15.193 | 9.843 | 20.543 | 2.730 | **< 0.0001** | 0.494 |
| **Anti-Ribosome %** | 2.396 | -8.849 | 13.640 | 5.737 | 0.676 | 0.434 |
| **Anti-tRNA synthetase %** | 1.775 | 0.061 | 3.511 | 0.890 | **0.046** | **0.012** |
| **Ethnicity** |  |  |  |  | **< 0.0001†** | 0.129† |
| Asian (Ref*) | _ | _ | _ | _ | _ | _ |
| American | -0.3831 | -0.5557 | -0.2105 | 0.0881 | **< 0.0001** | 0.266 |
| Caucasian | -0.4055 | -0.5313 | -0.2797 | 0.0642 | **< 0.0001** | 0.113 |
| **Context** |  |  |  |  | **< 0.0001†** | **< 0.0001†** |
| Broad requests (1.4%) (Ref) | _ | _ | _ | _ | _ | _ |
| Suspected CTD (8.1%) | 0.1697 | 0.1109 | 0.2286 | 0.0300 | **< 0.0001** | **< 0.0001** |
| Diagnosed CTD (44.1%) | 0.5683 | 0.5066 | 0.6299 | 0.0315 | **< 0.0001** | **< 0.0001** |
| **ANA detection** |  |  |  |  |  |  |
| HEp-2000 (Ref) | _ | _ | _ | _ | _ | _ |
| HEp-2 | 0.1960 | 0.0477 | 0.3443 | 0.0757 | **0.005** | **0.033** |
| **ENA subtyping method** |  |  |  |  | 0.156† | 0.189† |
| DID | _ | _ | _ | _ | _ | _ |
| Dot-blot/Immunoblot | 0.3266 | -2.0498 | 2.7029 | 1.2125 | 0.394 | 0.253 |
| ELISA | -1.3764 | -3.8154 | 1.0626 | 1.2444 | 0.134 | 0.414 |
| FEIA | 0.6847 | -2.2722 | 3.6416 | 1.5087 | 0.325 | 0.369 |
| MFIA | 1.4363 | -1.1572 | 4.0298 | 1.3232 | 0.139 | 0.184 |

*Ref: Reference category, †overall *p*-value
